# Actn4 Links Inactive Integrin α5 With Actin in Zebrafish Somites

**DOI:** 10.1016/j.mcpro.2025.101087

**Published:** 2025-10-08

**Authors:** Guangyu Sun, Scott A. Holley

**Affiliations:** 1 Xiamen Cardiovascular Hospital of Xiamen University, School of Medicine, Fujian Branch of National Clinical Research Center for Cardiovascular Diseases, Xiamen, Fujian, China; 2 Department of Molecular, Cellular and Developmental Biology, Yale University, New Haven, Connecticut, USA

## Abstract

Integrins are key plasma membrane proteins that mediate cell-ECM adhesion and communication, and they rely on a conformational change for their activation and bidirectional signaling. However, there are few *in vivo* studies of integrin activation. Here, we identify Integrin α5 (Itgα5)-associated proteins in the physiological setting of zebrafish somite morphogenesis. Using label-free mass spectrometry, we compared Itgα5-associated proteins in different integrin activation states. As expected, we found active Itgα5 enriched extracellular matrix (ECM) proteins. Surprisingly, inactive Itgα5 incapable of binding ligand recruits actin cytoskeletal proteins as efficiently as the active integrin. We validated Itgα5's linking to actin adaptors using Parallel Reaction Monitoring (PRM). We then focused on α-actinin 4 (Actn4), an actin cross-linker, which we find preferentially associates with inactive Itgα5. Along zebrafish somite boundaries, Itgα5 and Actn4 displayed on and off co-localization, and Actn4 showed a stronger correlation with wild-type and inactive Itgα5 compared with the active Itgα5. We also found that deleting the actin-binding domain (Actn4^ABDdel^) resulted in cytoplasmic retention and loss of colocalization with Itgα5. These findings suggest that Itgα5 and Actn4 cooperate during somite boundary formation and that actin cytoskeleton reorganization facilitates their colocalization. Furthermore, we showed ligand-binding-deficient Itgα5 associated with Paxillin a (Pxna), a scaffold protein highly enriched at somite boundaries and strongly correlated with activated Itgα5. This study provides novel insights into *in vivo* integrin activation and integrin-actin interactions and broadens our understanding of integrin’s role in tissue morphogenesis. Data are available via ProteomeXchange with identifiers PXD024942, PXD065495, PXD058516, PXD058550, and PXD058747.

## Highlights

• Explore Itgα5 activation-state-dependent associated proteins in zebrafish embryos.
• Inactive Itgα5 recruits actin cytoskeletal proteins.
• α-actinin 4 (Actn4), an actin cross-linker, preferentially associates with inactive Itgα5.
• Dynamic co-localization of Itgα5 and Actn4 during zebrafish somite boundaries formation.
• Actin cytoskeleton reorganization may facilitate Itgα5 and Actn4 contacts.

## Graphical Abstract

**Figure:**
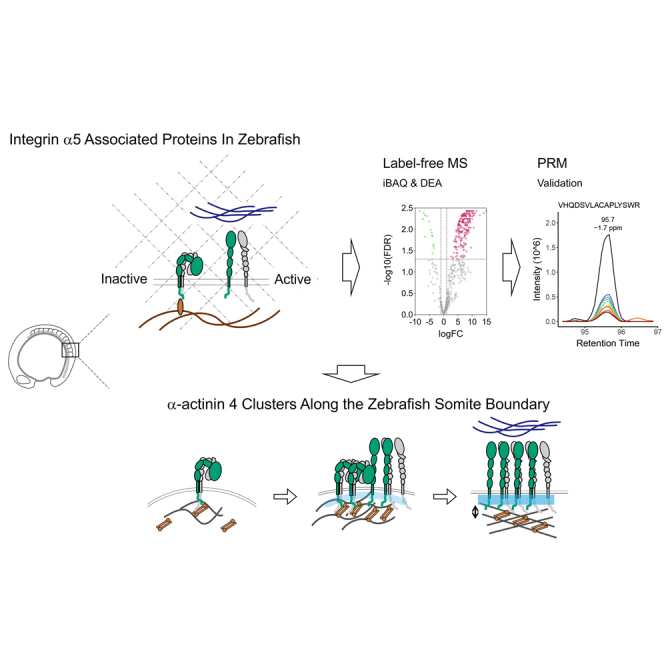

## In Brief

We compared associated proteins with active and inactive Itgα5 in zebrafish embryos using label-free mass spectrometry and validated results using PRM. Ligand-binding-deficient Itgα5 maintained connections with actin cytoskeletons and Actn4 emerged as a key player. Contacts between Itgα5 and Actn4 along the somite boundary were highly dynamic with a tendency to correlate strongly before Itgα5 is fully activated.

Integrins are αβ heterodimeric transmembrane proteins that link the extracellular matrix (ECM) to the cytoskeleton. They function in cell adhesion, migration, and morphogenesis, and thus are key regulators in various physiological and pathological processes, such as embryonic development, immune responses, and cancer (1, 2, 3, 4). Integrin activation involves a conformational change that increases binding affinity for its ligands, and this activation can be responsive to both intracellular and extracellular signals (5). Therefore, understanding the mechanisms of integrin activation is important for understanding their roles in physiology and disease.

Intracellular activation of integrins via their cytoplasmic domains, called inside-out signaling, is mediated by adaptor proteins such as Talin that link integrins to the actin cytoskeleton (6, 7, 8). Talin is critical in integrin activation and signaling, as integrins lack direct actin-binding ability. Focal adhesions (FA) in cell migration have been the most intensively studied cellular context for understanding these adaptors’ role in integrin activation (9, 10, 11, 12). Talin binds to integrin cytoplasmic tails and promotes integrin conformational changes to the activated state with higher affinity for extracellular ligand (13). Additionally, Talin regulates integrin adhesion strength and FA size in response to mechanical forces (8). Decades of work have revealed several modules mediating integrin activation and the context-dependent function of the ECM-integrin-cytoskeleton network, such as ILK–PINCH–parvin as cell cortex adaptors and Talin-Kindlin-Vinculin as the core actin–integrin linkage (14).

Integrin α5 heterodimerizes with Integrin β1 and is a key player in FA formation and ECM assembly. It functions as the primary fibronectin (FN) receptor, recognizing its Arg-Gly-Asp (RGD) domain (5, 15). While other integrins like Integrin αVβ3, α3β1, or α2β1 initiate focal contacts, Integrin α5β1 clustering promotes FA maturation and fibronectin fibrillogenesis (16, 17). Moreover, a recent study showed Integrin α5β1 nanospacing regulates collective cell migration in a substrate rigidity-independent manner (18). In signal transduction, Integrin α5β1 enhances RhoA activity, leading to subsequent cytoskeletal reorganization (19, 20). In mechanotransduction, it is activated by forces from both cell contraction and increased ECM stiffness (21, 22). Integrin α5β1 forms a catch-bond with fibronectin, which strengthens cell-ECM adhesion (23, 24).

*In vitro* studies provide more controlled experimental systems to study integrin biology, but discordance between 2D cell culture and the 3D physiological data underscores the need to also explore integrin regulation *in vivo* (10, 12). Integrin α5β1 is important in embryo development, especially somitogenesis (25, 26, 27, 28). Somites are segmented mesodermal structures that form sequentially in a head-to-tail progression along the embryonic axis in bilaterally symmetric pairs flanking the neural tube. In zebrafish somite morphogenesis, Integrin α5β1 along the nascent somite boundary is activated through conformational changes, facilitates fibrillar matrix formation, and finally establishes tissue boundaries (26, 29). Our previous work with this system revealed two key findings overlooked in cell culture studies: cadherin's inhibitory effect on Integrin α5 (29) and an inverse correlation between the intra-heterodimer stability and integrin activatability (30). These insights highlight the value of studying integrins in more complex native environments.

In this study, we extended our investigation into Integrin α5 (Itgα5) activation during zebrafish somitogenesis. We compared Itgα5-associated protein networks in inactive and active states using mass spectrometry. Characterization of these activation-state-dependent proteins led to a focus on the ECM-integrin-actin axis, specifically the actin adaptors. Surprisingly, ligand-binding-deficient Itgα5, considered inactive, preserved connections with actin. α-actinin 4 (Actn4) emerged as a key player in linking Itgα5 and actin, especially before Itgα5 is fully activated. We characterized colocalization dynamics between Itgα5 and Actn4 during somite boundary formation. Our comprehensive analysis will be a valuable resource for identifying *in vivo* Itgα5 regulators and effectors. This study also provides new insights into the integrin–actin interactions and broadens our understanding of integrin regulation *in vivo*.

## Experimental Procedures

### Experimental Design and Statistical Rationale

This study included three sets of experiments: (1) integrin α5 (Itgα5) activation-state-dependent proteomics experiments and bioinformatics analysis, (2) protein of interest validation using Parallel Reaction Monitoring (PRM), and (3) examination of α-actinin 4 (Actn4) as an Itgα5-actin adaptor during zebrafish somitogenesis.

For the first set of experiments, we collected Itgα5 activation-state-dependent associated proteins at the zebrafish 14 somite stage. Ligand-binding-deficient mutant allele (Itgα5^LBD^) with five amino acid substitutions that eliminate both the RGD and synergy site binding represents the inactive state while Itgα5^AA^ has two amino acid substitutions in the membrane proximal cytoplasmic domain that eliminates a salt-bridge and is constitutively active (6, 31, 32). The two integrin alleles were tagged with green fluorescent protein (GFP) and the FLAG epitope. *In vitro* transcribed mRNAs encoding the alleles were microinjected into embryos at the 1-cell stage at the minimal concentration required for the wild-type allele to rescue the *itgα5* mutant phenotype. The GFP label enabled screening of embryos for consistent ubiquitous expression, and the FLAG tag was used for co-immunoprecipitation of Itgα5 associated proteins. Three biological replicates, also the technical replicates, in each state were sent for further proteomic analysis. The proteomic profiles of the inactive and active integrins were compared. Wild-type Itgα5-GFP-FLAG and membrane anchored mem-GFP-FLAG as negative control were examined in the same experiments and reported in a prior study (30). These controls are used for data analysis in this study. The number of replicates was selected as appropriate accounting for experimental variation and the stochastic nature of MS/MS sampling.

In the second set of experiments (validation), we prepared the samples in the same manner as in the first set of experiments. Mem-GFP-FLAG was used as a negative control. Three biological replicates for each state and two biological replicates for the negative control were processed for PRM experiments.

In the last set of experiments, we focused on Actn4, an actin cross-linker, characterized its expression and co-localization with Itgα5 during the zebrafish somite boundary formation, and explored their association on a molecular level. At least three embryos were used for each imaging experiment.

### Zebrafish Maintenance

Zebrafish were maintained in accordance with standard protocols approved by the Institutional Animal Care and Use Committee at Yale University (IACUC) and the Animal Care and Use Committee at the School of Medicine, Xiamen University. Wild-type strains used are TLF. The zebrafish resources TLF (CZ2) used in this study at Xiamen University were provided by the China Zebrafish Resource Center, National Aquatic Biological Resource Center, CZRC/NABRC). The MZItgα5^−/−^ mutant line is a maternal zygotic mutant line using the *tbfe1* (e.g*. thl030*) allele (33).

### Fluorescent Protein Constructs and In Vitro Transcription

All fluorophores were tagged at the C-terminus with a two amino acid spacer between integrin and fluorophore. The vector used was pCS2+. The GFP variant used was emerald GFP, and red fluorescent protein (RFP) was tagRFP. Intracellular myristoylated membrane-anchored negative control mem-GFP, Itgα5-GFP-FLAG, Itgα5-RFP, Itgα5^FYLDD^-GFP, and Itgα5^GAAKR^-GFP constructs were previously described (31). For co-immunoprecipitation experiments, C-terminal epitope FLAG (34) tagged integrin-GFP constructs were generated. α-actinin 4 (Actn4, UniprotID: Q7SYE2) coding sequence was amplified via PCR from 16 to 25 h post fertilization (hpf) cDNA generated from the TLF strain and cloned into pCS2+ vector. The PCR primers were F: ATGGTAGATTATCACGCCGTT and R: GAGGTCGCTCTCCCCGTACAG. New plasmids were generated from PCR products of the protein coding sequence, fluorophores, and double digestion products of pCS2+ vector from available constructs using Gibson Assembly Master Mix (NEB, E2611). Paxillin a (Pxna, UniprotID: Q6R3L1, NCBI Reference Sequence: NM_201588.2) coding sequence was synthesized and cloned into pCS2+ vector (Sangon Biotech).

To create Actn4 mutant constructs, the sequence was first aligned with homo sapiens ACTN4 (UniprotID: O43707). In actin binding domain deleted (Actn4^ABDdel^) mutant, amino acids 39 to 262 were deleted (35). This mutant was generated from Actn4-RFP using overlap extension PCR. For the point mutations, Actn4^Y4/21E^ and Actn4^K245E^ (36, 37, 38), DNA fragments containing the mutation sequence were synthesized and cloned into the Actn4-RFP vector by available double digest sites or overlapping PCR (Sangon Biotech).

For mRNA synthesis, the respective plasmids were linearized with NotI-HF (NEB, R3189), the mRNA *in vitro* transcribed with the Sp6 mMessage mMachine kit (Invitrogen, AM1340) and cleaned with the Micro Bio-Spin Columns with Bio-Gel P-30 (Bio-Rad, 732–6250). mRNA was injected into one-cell stage embryos.

### Confocal Microscopy

#### Sample Preparation

Embryos at the 12 to 14 somite stage were manually dechorionated and embedded in 0.8% low-melt agarose (Sangon Biotech, A600015) in a glass-bottom dish with a thickness of No. 1.5 (NEST, 801021). The dorsal side of the embryo faced the cover glass. Experiments were performed at room temperature (22 °C).

#### Image Acquisition

Acquisition of confocal imaging was performed on Leica Stellaris 5 Wll confocal microscope using an HCX PL APO 63×/1.40 oil objective. Excitation was provided by the White Laser 488 nm and White Laser 561 nm. The laser power measured before the objective was 30 μW. Images were acquired in the Leica Lightning mode with adaptive deconvolution with 1024 × 1024 resolution, line sequential, and line average of 2. Time-lapses were taken at 3-min intervals.

#### Image Quantification

Along the somite boundary, marked by a cleavage seen under transmitted light, the green channel (integrin) was used as a reference to select a region of interest (ROI) by drawing a line (Fig. 4). For each channel as the reference channel, intensity readings were taken at top 5, 10, 20, 30, 40, 50, 60, 70, 80, 90, and 100%, Spearman's Rank Correlation Coefficient (*r*_*s*_) was calculated between the intensities (Int) of the extracted pixels of the reference channel and the corresponding pixels of the other channel using R's function: cor(Int_green_, Int_red_, method = "spearman", use = "complete.obs"). Pixels with an intensity reading of 0 were excluded from the calculation, along with their corresponding pixels in the other channel. For co-localization tracking (Supplemental Figs. S7 and S8), boundaries were drawn based on green and red channels separately, and the correlation coefficients were calculated between the reference channel and the matching pixels from the other channel. Results of *r*_*s*_ using all pixels are listed in Supplemental Table S14.

### Proteomic and Bioinformatic Analyses

**Fig. 1:**
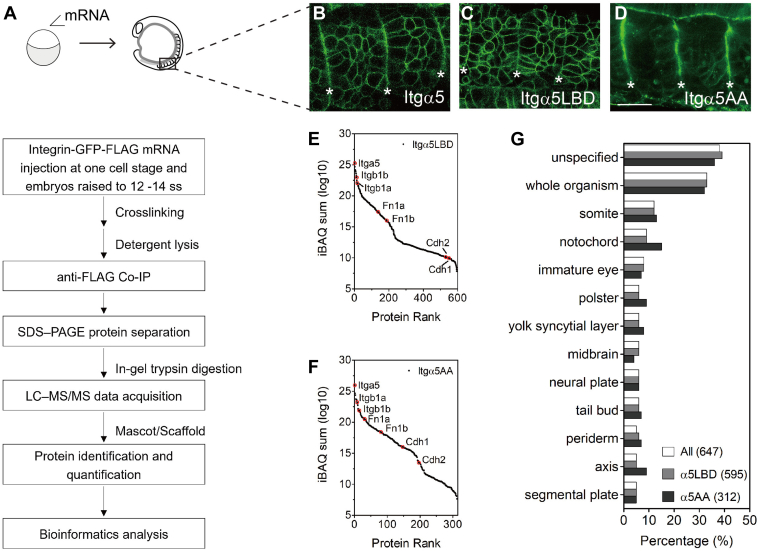
**Activation-state-dependent Integrin α5 associated proteins in zebrafish somites.***A*, affinity-based mass spectrometry workflow. ss, somite stage. *B–D*, expression of GFP-FLAG labeled Integrin α5 (Itgα5, *B*) and its two mutant alleles, ligand-binding-deficient Itgα5LBD (*C*) and constitutively activated Itgα5AA (*D*), in early zebrafish somites. *Asterisks* indicate somite boundaries. Scale bar: 25 μm. *E*–*F*, protein abundance rank in Itgα5LBD (*E*) and Itgα5AA (*F*) datasets. *Red circle*: representative proteins with protein symbols shown. *G*, gene anatomies distributions at the experimental stage. The results are listed in supplemental Tables S1 and S4.

The overall workflow of proteomic and bioinformatic analyses was depicted in Figure 1*A*.

#### Sample Preparation

Samples were prepared according to previous publication (30). MZItgα5^−/−^ embryos were used to eliminate endogenous unlabeled Integrin α5. Injection of wild-type Itgα5 rescues the mutant phenotype of these embryos (31). Briefly, for each replicate, about 120 embryos were injected with mRNA (250 ng/μl, 450 pg) encoding GFP-FLAG-tagged integrins at the one-cell stage, raised to 12 to 14 somite stage, and then dechorionated using Pronase (Sigma). In the mem-GFP-FLAG control, injected mRNA concentration was reduced to achieve green fluorescent brightness similar to that of Itgα5s in confocal fluorescence imaging. After rinsing with modified Ringer’s solution (116 mM NaCl, 3 mM KCl, 4 mM CaCl_2_, 1 mM MgCl_2_, 5 mM HEPES pH 7.8), embryos were subjected to a crosslinking treatment by incubation in modified Ringer’s solution containing 5 mM DTBP (Dimethyl-3,3′-Dithiobispropionimidate, Thermo Scientific) at 28.6 °C for 5 h. Note that this crosslinking treatment was employed to capture transiently or weakly associated proteins, rather than for crosslinking mass spectrometry (XL-MS) analysis. Then, the crosslinking reaction was quenched by incubating in modified Ringer’s solution containing 50 mM Tris-HCl pH 7.6 on ice for 20 min. Embryos were then transferred into 200 to 400 μl lysis buffer (50 mM Tris pH7.6, 150 mM NaCl, 1 mM EDTA, 10% glycerol, one tablet cOmplete protease inhibitor cocktail, 5% Triton X-100, 0.1% IGEPAL), disrupted manually in Eppendorf tubes with a pestle (Fisherbrand), incubated on ice 30 min with gentle vortexing every 5 min, and clarified by 10 min centrifugation at 10,000*g*. The supernatant was transferred to fresh tubes and kept on ice before immunoprecipitation.

#### Immunoprecipitation (IP)

The anti-FLAG M2 affinity gel (A2220, Sigma) was prepared according to the manufacturer’s instructions. Briefly, 20 μl packed gel per sample was prepared by washing three times briefly in 400 μl TBS, once for 5 min in 500 μl 0.1 M glycine pH 3.5, four times in 400 μl TBS. All centrifugation was at 7000*g* except the last two wash steps which were at 10,000*g*. Samples were exposed to affinity gel overnight at 4 °C with gentle agitation. Immunoprecipitates (IPs) were washed four times in 500 μl TBS. After washes, 35 μl 2× Laemmli Sample Buffer (Bio-Rad) was added to affinity resins, and the mixture was incubated at 95 °C for 7 min, followed by incubation on ice for 1 min and centrifugation for 30 s at 8200*g*. The supernatant (about 30 μl) was transferred to a fresh tube and kept at 4 °C or −20 °C (for longer storage) until running on 10% sodium dodecyl sulfate-polyacrylamide gel (SDS-PAGE).

#### Coomassie Staining

Following SDS-PAGE, total protein was visualized by incubating gels in Coomassie staining solution (0.1% (w/v) Coomassie Brilliant Blue G 250 (AmericanBio), 10% (v/v) acetic Acid, 45% (v/v) methanol) for 2 h at room temperature. Gels were then destained in destaining buffer (10% (v/v) Acetic Acid, 20% (v/v) Methanol). Between each step, the gel was washed with excess distilled H_2_O. After destaining, lanes were sliced into 2 slices, higher than 75 kDa and 25 to 75 kDa. Samples were kept at −20 °C before being sent for MS analysis. Representative Coomassie staining gels are shown in supplemental Fig. S2, and a summary of the sample amounts used in all MS experiments is documented in supplemental Table S13.

#### In-gel proteolytic digestion

Gel slices were cut into small pieces and washed for 10 min with water, followed by washing for 30 min with 1 ml 50% acetonitrile (ACN)/100 mM NH_4_HCO_3_ (ammonium bicarbonate, ABC). The samples were reduced by the addition of 80 μl 4.5 mM dithiothreitol (DTT) in 100 mM ABC with incubation at 37 °C for 30 min. The DTT solution was removed, and the samples were cooled to room temperature. The samples were alkylated by the addition of 80 μl 10 mM iodoacetamide (IAN) in 100 mM ABC with incubation at room temperature in the dark for 30 min. The IAN solution was removed, and the gels were washed for 15 min with 900 μl 50% ACN/100 mM ABC and then washed for 15 min with 900 μl 50% ACN/25 mM ABC. The gels were briefly dried by SpeedVac, then resuspended in 80 μl of 25 mM ABC containing 400 ng of digestion grade trypsin (Promega, V5111) and incubated at 37 °C for 16 h. The supernatant containing tryptic peptides was transferred to a new Eppendorf tube, and the gel band was extracted with 350 μl of 80% acetonitrile/0.1% trifluoroacetic acid (TFA) for 15 min. Supernatants were combined and dried by speed vacuum. Peptides were dissolved in 25 μl MS loading buffer (2% ACN, 0.2% TFA), with 5 μl injected for LC-MS/MS analysis.

#### Liquid Chromatography with Tandem MS Analysis

LC-MS/MS analysis was performed on a Thermo Scientific Q Exactive Plus equipped with a Waters nanoAcquity UPLC system utilizing a binary solvent system (A: 100% water, 0.1% formic acid; B: 100% acetonitrile, 0.1% formic acid). Trapping was performed at 5 μl/min, 99.5% Buffer A for 3 min using a Waters ACQUITY UPLC M-Class Symmetry C18 Trap Column (100 Å, 5 μm, 180 μm × 20 mm, 2G, V/M). Peptides were separated at 37 °C using a Waters ACQUITY UPLC M-Class Peptide BEH C18 Column (130 Å, 1.7 μm, 75 μm × 250 mm) and eluted at 300 nl/min with the following gradient: 3% buffer B at initial conditions; 5% B at 2 min; 25% B at 140 min; 40% B at 165 min; 90% B at 170 min; 90% B at 180 min; return to initial conditions at 182 min. MS was acquired in profile mode over the 300-1700 m/z range using 1 microscan, 70,000 resolution, AGC target of 3E6, and a maximum injection time of 45 ms. Data-dependent acquisition (DDA) MS/MS were acquired in centroid mode on the top 20 precursors per MS scan using 1 microscan, 17,500 resolution, AGC target of 1E5, maximum injection time of 100 ms, and an isolation window of 1.7 m/z. Precursors were fragmented by HCD activation with a collision energy of 28%. MS/MS were collected on species with an intensity threshold of 1E4, charge states 2 to 6, and peptide match preferred. Dynamic exclusion was set to 20 s.

#### Label-Free Peptide and Protein Identification

Tandem mass spectra were extracted by Proteome Discoverer software (version 2.2.0.388, Thermo Scientific) and searched in-house using the Mascot algorithm (version 2.6.1, Matrix Science). The data were searched against a Uniprot reference proteome for *Danio rerio* (Uniprot_Danre_v190523_46927ent). Search parameters included trypsin digestion with up to 2 missed cleavages, peptide mass tolerance of 10 ppm, and MS/MS fragment tolerance of 0.02 Da. Cysteine carbamidomethylation and methionine oxidation were configured as variable modifications. Normal and decoy database searches were run, with the confidence level set to 95% (*p*-value <0.05). Scaffold (version 4.9.0, Proteome Software Inc., Portland, OR) was used to validate MS/MS-based peptide and protein identifications. Peptide identifications were accepted if they could be established at greater than 95.0% probability by the Scaffold Local FDR algorithm. Protein identifications were accepted if they could be established at greater than 99.0% probability and contained at least 2 identified peptides. Protein probabilities were assigned by the Protein Prophet algorithm (39). Proteins that contained similar peptides and could not be differentiated based on MS/MS analysis alone were grouped to satisfy the principles of parsimony. Proteins sharing significant peptide evidence were grouped into clusters. The cluster representative was used for further quantification. Protein and peptide identification results are listed in supplemental Tables S1 and S2.

#### Label-Free Protein Quantification and Statistical Analysis

**Fig. 2:**
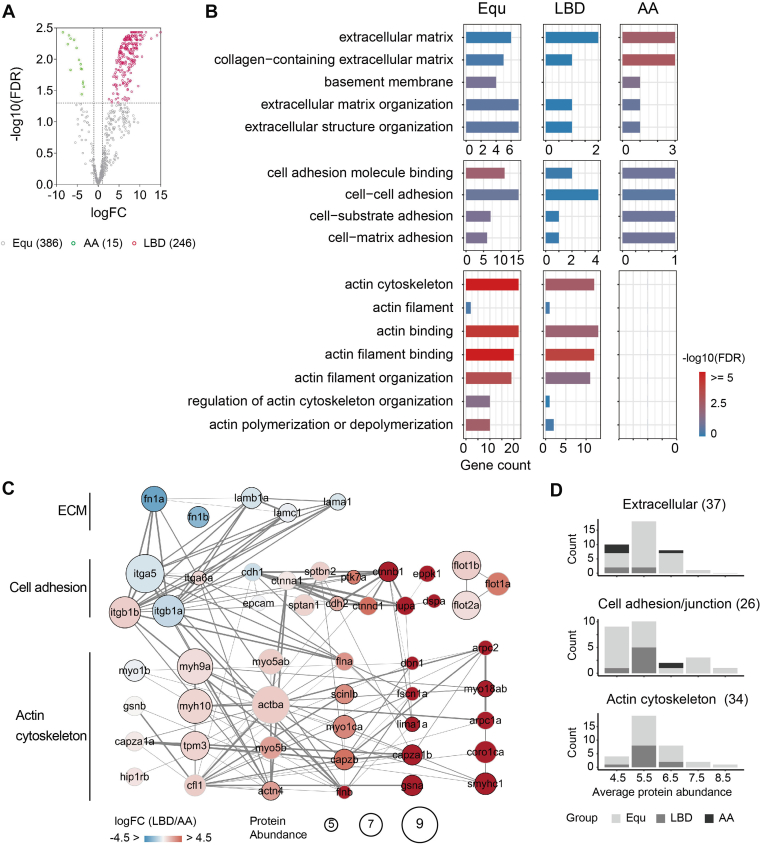
**Ligand-binding-deficient Integrin α5 connects with actin cytoskeleton.***A*, Volcano plot of the differential expression analysis between the Itgα5-associated proteins at two activation states. FC: fold change; FDR: false discovery rate. *Red* circle, group LBD, Itgα5LBD enriched; *green* circle, group AA, Itgα5AA enriched; *grey* circle, group Equ, equally shared in the two datasets. *B*, overrepresentation analysis of the representative ECM and actin cytoskeleton GO terms by groups. Color code indicates FDR. *C*, ECM-integrin-actin network. Gene names are used for illustration. Node size: protein abundance in log10 transform; node color: fold change (FC); edge thickness: interaction score; node with *black* circle: gene expression in somite area. *D*, average protein abundance by groups. Results are listed in supplemental Tables S3–S6.

For label-free quantification, the intensity-based absolute quantification (iBAQ) algorithm (40, 41) from Scaffold was used. iBAQ divides the raw intensities by the number of theoretical peptides. The intra-experiment correlation displayed Pearson's correlation coefficient values of 0.92 and 0.59 in the good and poor groups, respectively, and an average of 0.78 (supplemental Fig. S1). Proteins appeared in at least two replicates were processed with missing values (if any) replaced by the median of the non-missing values. Other missing values were assigned as the minimum within the replicate. For comparisons to the negative control, we performed the one-sided (greater) *t* test with a significance level *p*-value <0.1. Proteins not significantly more abundant in any datasets compared to the negative control dataset were discarded. To compare Itgα5 associated proteins at different activation states (Fig. 2), the differential expression analysis was performed using the R Package limma (version 3.60.5). The significance level was set as adjusted *p*-value <0.05 and fold-change (logFC) > 1. Proteins with fold-change (logFC) > 1 and adjusted *p*-value <0.05 were assigned to group "LBD"; fold-change (logFC) < −1 and adjusted *p*-value <0.05 were assigned to group "AA"; others to group "Equ". Results are listed in supplemental Table S3. To compare the two Itgα5 mutants with the previously reported wild-type Itgα5 (supplemental Fig. S3), the hierarchy clustering analysis was performed based on Euclidean distances and complete linkage matrix. Clustering results are appended in supplemental Table S1. Results were visualized using the R pheatmap package (version 1.0.12).

#### Gene Anatomy

Gene expression was annotated using recordings from ZFIN (https://www.zfin.org/downloads/wildtype-expression_fish.txt, downloaded date: 2023.06.25). Superstructures that begin to form before 18 h post fertilization (hpf) and are present by at least 10 hpf were selected, considering that somite begins around 10 hpf and our experimental stage was around 16 hpf. Tissues or organs like the liver, which display gene expression but develop much later, were excluded from the list. Results are recorded in supplemental Table S4 and plotted in Figure 1*G*.

#### Gene Ontology Annotations

Bioconductor (42, 43) was used for the gene ontology annotation. The org.Dr.eg.db package (version 3.19.1) was used for ENTREZID retrieving and the gene ontology annotation. Full GO annotation results are appended in supplemental Table S4. The clusterProfiler package (version 4.12.6) was used for the enrichment analysis and the statistical significance was p.adjust <0.05. Results are listed in supplemental Table S5 for Figure 2*B* and supplemental Fig. S3, *D* and *E*, and supplemental Table S7.2 for supplemental Fig. S4, *C*–*F*.

#### Adhesome and Consensus

Integrins are the fundamental components of cell adhesion. The adhesome, a protein-protein interaction (PPI) network comprised of cellular components of the focal adhesion complex in mammalian cells, demonstrated a well-defined integrin-centred network (9, 44). Using Fibronectin as the substrate, a meta-analysis also summarizes a consensus list (11). To crosscheck our integrin-associated proteins with the adhesome components, we retrieved the human orthologs from the “Human and Zebrafish Orthology” document from ZFIN (downloaded date: 2023.02.03). The adhesome dataset was downloaded from https://adhesome.org/. The consensus dataset was supplementary Table S4 in Horton’s work (11). Results are appended in supplemental Table S4.

#### ECM-Integrin-Actin Networks

We explored integrin centered ECM-integrin-actin networks in Figure 2*C*. For extracellular proteins, we included proteins from GO terms, extracellular region (GO:0005576), extracellular space (GO:0005615), and extracellular matrix (GO:0031012). The top abundant ECM proteins, fibronectins (Fn1a and Fn1b) and laminins (Lama1, Lamb1a, Lamc1), are shown. For actin cytoskeleton proteins, we included proteins from GO terms, actin cytoskeleton (GO:0015629), actin binding (GO:0003779), and actin cytoskeleton organization (GO:0030036). We also included the cell-cell adhesion/junction proteins from GO terms, cell-cell adhesion (GO:0098609), cell-cell junction (GO:0005911), cell junction (GO:0030054), protein localization to cell-cell junction (GO:0150105), and positive regulation of cell junction assembly (GO:1901890). PPI networks were searched using the R package STRINGdb (version 2.16.4) against “7955.protein.links.v12.0.txt.gz” with default settings. The network was then imported to Cytoscape (Version: 3.8.2) for visualization. The node size was the sum of iBAQ readings (log10 transform) in every dataset to indicate protein abundance, the larger the node, the more protein detected in total. The node color was the logFC, the warmer the color, the more likely the protein was enriched in the LBD group, and the colder, the more likely it was enriched in the AA group. The edge thickness was coded by the interaction score (combined_score), the thicker indicated the stronger evidence of the interaction. Nodes with black circles indicated gene expression in the posterior embryo, including somite (ZFA:0000155), segmental plate (ZFA:0000279), notochord (ZFA:0000135), and tail bud (ZFA:0000077). The interactions and scores are listed in supplemental Table S6.

#### Itgα5LBD’s Interactome

Protein abundance classification was based on quantitative ranking, with “high” representing proteins above the 75th percentile, “low” below the 25th percentile, and “medium” between the 25th and the 75th percentiles. Subcellular distribution comparisons between these classes and the whole dataset (all) were performed using Fisher's exact test using the R function fisher.test, and results are listed in supplemental Table S7.1 and plotted in supplemental Fig. S4*B*. The PPI network was constructed from actin cytoskeletal and ribosomal components in GO terms that were found to be significantly enriched in quantity classes (supplemental Tables S7.2 and S7.3). Interaction searching and plot setting followed the same scheme as in the ECM-Integrin-Actin networks, except that the node size was the median of three replicates in log10 transform. The network centrality was calculated using the degree function from the R package igraph (version 2.0.3).

### Development and Analytical Validation Targeted MS Assays

**Fig. 3:**
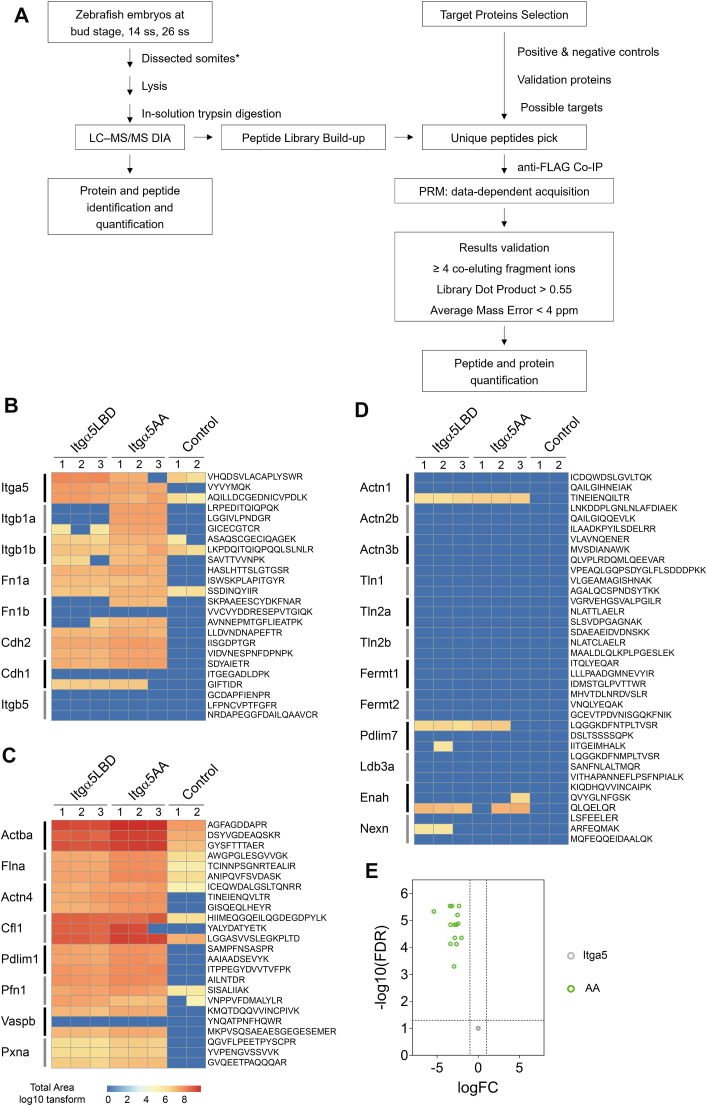
**Results validation by parallel reaction monitoring (PRM).***A*, PRM experiment workflow. ss, somite stage. Somite dissection was performed for 14 and 26 ss embryos. *B*, Itgα5 and the known binding partners as positive control. Itgβ5 served as the negative control. *C*, detected integrin-actin adaptors. *D*, undetected integrin-actin-adaptors. *B–D*, each line represents a unique peptide sequence with sequence on the *right* and target protein on the *left*; cell color: total area of the precursor in log10 transform; protein symbols are used for illustration. *E*, differential expression analysis of protein quantification. Results are in supplemental Table S11 and S12.

The targeted proteomic assays reported in this study are Tier 3 level, which aims to detect a predefined set of analytes. The overall workflow was depicted in Figure 3*A*.

#### Peptide Library Build-up

To identify the protein and peptide candidates for Parallel Reaction Monitoring (PRM), we first performed Data Independent Acquisition (DIA) mass spectrometry on the total protein extracts from zebrafish embryo somites. To maximize the coverage and confidence of protein identification, we also included developmental stages before and after the 14 somite stage, that is, the bud stage and the 26 somite stage. 20 embryos per sample were dechorionated using Pronase (Sigma) and washed four times with cold E2 buffer (7.5 mM NaCl, 0.25 mM KCl, 0.5 mM MgSO_4_, 75 μM KH_2_PO_4_, 25 μM Na_2_HPO_4_, 0.5 mM CaCl_2_, and 0.35 mM NaHCO_3_). For 14 and 26 somite stage embryos, following incubation on ice for 10 min, somites were dissected in a glass holder on ice and collected in pre-cooled E2 buffer. Samples were then processed using a modified deyolking procedure (45). Briefly, samples were washed with cold deyolking buffer (55 mM NaCl, 3.6 mM KCl, and 1.25 mM NaHCO_3_) with inverting followed by centrifugation at 13,000 RPM for 1 min at 4 °C, and then washed with cold wash buffer (10 mM Tris-HCl, pH 7.4) with quick vortex followed by centrifugation at 13,000 RPM for 1 min at 4 °C. Tissue pellets were disrupted manually in Eppendorf tubes with a pestle (Fisherbrand) and lysed in SDS sample buffer (63 mM Tris-HCl pH 6.8, 10% (v/v) glycerol, 3.5% (v/v) SDS). The lysates were incubated at 95 °C for 10 min with gentle agitation (500 RPM), cooled on ice, and centrifuged at 13,000 RPM for 20 min at 4 °C. The supernatant was collected and kept at −80 °C. To ensure compatibility with DIA analysis, samples were dried using the Liquid Sample Total Protein Extraction Kit (Applygen, P1255) to remove detergent and resuspended in 8 M Urea. Protein concentration was then determined using the Pierce BCA Protein Assay Kit (Thermo Scientific, 23225). 20 micrograms of protein from each sample were sent for DIA analysis.

Protein samples were subjected to in-solution trypsin digestion and dried. Briefly, proteins were reduced by 25 mM tris (2-carboxyethyl) phosphine and alkylated by 50 mM chloroacetamide and then separated on a NuPAGE 4 to 12% BisTris gel (Life Technologies). Protein bands were visualized by Coomassie blue. Full gel region was excised and the in-gel digestion was performed using trypsin (signalchem, T575–31N-1000). Peptides were desalted using C18 stage tips, concentrated to near dryness by vacuum centrifugation, and resuspended in 1% formic acid (10 μl). Peptide samples were then analyzed on an EASY-nLC 1200 (Thermo SCIENTIFIC) coupled to an Orbitrap Fusion Lumos (Thermo SCIENTIFIC) equipped with an EASY-IC ion source. Peptides were auto-sampled directly onto a homemade C18 column (35 cm × 75 μm i.d., 2.5 μm 100 Å). Samples were then eluted for 120 min with linear gradients of 3 to 35% acetonitrile in 0.1% formic acid at a flow rate of 300 nl/min. For DIA, a 25 m/z precursor isolation width covering 400 to 1200 m/z was adopted.

The DIA files were generated using DIA-NN software133 (V.1.8.1) against a Uniprot reference proteome for *D. rerio* database, uniprot-Danio_rerio (Zebrafish)_7955_unreviewed_20230707 without decoys, during which the parameters were set as: “FASTA digest for library-free search” and “Deep learning-based spectra and RTs prediction” were enabled; Protease, “Trypsin/P”; Missed cleavage, “2”; N-termM excision, “checked”; c carbamidomethylation, “checked”; Moxidation, “checked”. The maximum mass accuracy tolerances were set to 10 ppm for both MS1 and MS2 spectra. Protein inference in DIA-NN was the protein name (from fasta). Quantification mode was set to “Robust LC (high accuracy)”. All other settings were left default. PG.MaxLFQ was used for protein quantification. Proteins with a minimum of two unique peptides detected and a PG.Q.Value smaller than 0.01 were included in further analysis. The full peptide and raw protein results are uploaded in PXD058516. The peptides of the target proteins and organized protein quantification results are listed in supplemental Tables S8 and S9. The constructed peptide library was used for PRM data analysis.

#### Target Proteins Selection

We first included as positive controls the proteins known to bind Itgα5. These proteins were Itgα5 heterodimer beta subunits, Itgβ1a and Itgβ1b, ECM ligands Fn1a and Fn1b, the weakly associated protein Cdh2 detected by Fluorescence Cross-Correlation Spectroscopy (29), and possibly Cdh1. We used Integrin β5 (Itgβ5) as a negative control. For validation, we then chose representative proteins in the integrin-actin networks (Fig. 2*C*), including Actba, Flna, Actn4, and Cfl1. Lastly, we picked several actin regulators and adaptors from the adhesome and consensus list. These proteins should first have *D. rerio* orthologs and then have a gene expression profile during zebrafish somitogenesis. In addition, their protein expression should be detected in the total protein extracts from zebrafish embryo somites at the 14 somite stage in DIA analysis. The candidates included Tln1, Fermt1, Fermt2, Pdlim1, Pdlim7, Ldb3a, Pfn1, Enah, Vaspb, Pxna, and Nexn. We included family members Actn1, Actn2b, and Actn3b for Actn4, our focus in this study. Note the isoform Actn3a was not on the list since it was not detected in the DIA experiment at the 14-somite stage. We also included family members Tln2a and Tln2b for Talin as they showed up once in the iBAQ results. Selection details are appended to supplemental Table S12.

#### Unique Peptide Sequence Selection

To obtain the unique peptide sequences for targeted proteins, we cross-checked the peptide sequences against the Uniprot reference (uniprot-compressed_true_download_true_fields_accession_2Creviewed_2C-2023.04.10-08.20.58.74.tsv.gz) to retain only the unique peptide sequences for our targets. To evaluate peptide uniqueness, gene names were used as the protein identifiers. Gene names were standardized using a hierarchical approach: the first "ordered locus gene name" was used as the primary identifier; when unavailable, the first "gene name" was used; in cases where neither was available, the UniProt entry ID was assigned. The stripped peptide sequences from the DIA results were then searched against the protein sequence list. The matching gene name(s) were recorded in the "Genes" column and the number of matches were recorded in "Gene.Hits". For proteins with identical sequences but different gene names, all gene names were recorded, but Gene.Hits was returned as 1. For peptides assigned to more than one protein, sequence similarity between proteins was assessed using the pairwiseAlignment function from the R package pwalign (v1.0.0). The sequence similarity between two protein sequences was calculated as the ratio of their pairwise alignment score to the self-alignment score of the first sequence. A peptide's tolerable uniqueness was TRUE if the assigned proteins shared ≥95% sequence similarity, for instance, Actba and Actbb (99.8%). The complete peptide uniqueness results of the DIA-MS dataset are uploaded in PXD058516 as "Stripped_Sequence_Uniqueness.Rdata". Note that two peptides (AGFAGDDAPR and DSYVGDEAQSKR) initially identified as unique to Actba were later found to be shared among multiple actin family members (Actc1a, Acta1b, Acta2, Actc2, Actb2/Actbb, Acta1a, Actc1b, Actba). To select the targeted peptides, we first ran the PRM experiment with the top 6 unique sequences based on the DIA intensities (MS1 Area) and then chose 3 sequences with the best performance for each target for the PRM.

#### Experiment

Samples were collected using the same protocol as for the LC-MS/MS with the following adjustments. The fish strain used was TLF. The eluted protein samples were run on 4 to 12% SDS-PAGE (LABLEAD P41212) and the total protein was visualized by incubating gels in LabBlue Coomassie brilliant blue quick staining solution (LABLEAD G1042) for 10 to 20 min at room temperature. Gels were then destained with excess distilled H_2_O for at least 4 h. After destaining, lanes were sliced into small pieces and kept in distilled H_2_O at 4 °C for fewer than 2 days before being sent for MS analysis. Representative Coomassie staining gels are shown in supplemental Fig. S2*C*. In PRM, the in-gel digestion was performed using trypsin (signalchem, T575-31N-1000). Peptides were desalted using C18 stage tips, concentrated to near dryness by vacuum centrifugation, and resuspended in 1% formic acid (10 μl). Samples were then analyzed on an EASY-nLC 1200 (Thermo SCIENTIFIC) coupled to an Orbitrap Fusion Lumos (Thermo SCIENTIFIC) equipped with an EASY-IC ion source. Peptide samples were auto-sampled directly onto a homemade C18 column (35 cm × 75 μm i.d., 2.5 μm 100 Å). Samples were then eluted for 120 min with linear gradients of 3 to 35% acetonitrile in 0.1% formic acid at a flow rate of 300 nl/min. The data-dependent acquisition raw files were analyzed by Proteome Discoverer 2.5 software against the Uniprot database (*D. rerio* uniprot-Danio_rerio (Zebrafish)_7955_unreviewed_20230707), to determine the m/z, z, and start/stop time of the candidate peptides. Further PRM parameters included an automatic gain control (AGC) of 1 × 10^5^, a maximum injection time of 1000 ms, and a precursor isolation window width of m/z 1. Skyline-daily 21.2.1.424 was used to analyze the PRM data.

#### Data Quantification and Statistical Analysis

Validation of the targeted peptides in the Skyline was performed by manual verification of peak picking. The DIA peptide library was used as the library in the skyline file for spectrum analysis. A candidate was considered validated if it was detected in ≥2 independent samples with ≥4 co-eluting fragment ions. Also, it should receive the Library Dot Product >0.55 and an Average Mass Error PPM between −4 and 4 ppm. The integration boundaries were manually validated. The summed Area values of all individual transitions for the particular precursor were used as peptide quantification. Proteins with at least two unique sequences detected and appeared in at least two replicates were kept. Missing values were handled in the same way for iBAQ data. The sum of the peptide amount was used as protein levels. To adjust differing Itgα5 levels in the two datasets (*p*-value = 0.003), protein abundances within each replicate were first normalized to their respective Itgα5 levels. Then the differential expression analysis was performed for protein quantification in the same way as for the iBAQ data (Fig. 3*E*). Peptide sequences and quantification results are listed in supplemental Tables S10 and S11. Protein quantification results are listed in supplemental Table S12.

### Statistical Analysis

Statistical analyses were performed using R language (version 4.4.0) and R Studio (version 2023.09.1). A *p*-value <0.05 was considered significant unless stated otherwise. Statistical details of experiments can be found in the figure legends. Comparisons were performed using an unpaired two-tailed *t* test unless stated otherwise.

## Results

### Integrin α5 Associated Proteins in Early Zebrafish Somites

To find Integrin α5 (Itgα5) partners, we compared the associated proteins in Itgα5’s inactive and active states using affinity-based mass spectrometry (Fig. 1*A*) (30). Ligand-binding-deficient-Itgα5 (Itgα5LBD) has an FYLDD mutation that eliminates the ability to bind either the RGD domain or the synergy site in Fibronectin (FN) and thus represents the inactive state of Itgα5 (31). Constitutively activated Itgα5 (Itgα5AA) has the GAAKR mutation, which removes the salt bridge between the integrin α and β subunits in membrane proximal cytoplasmic domain and leads to the open conformation and thus represents the active state of Itgα5 (6, 32). Here, we expressed green fluorescent protein (GFP) and FLAG epitope labeled Itgα5 mutants in zebrafish embryos by micro-injection at the one-cell stage and raised embryos to the 12 to 14 somite stage. To capture weakly and transiently associated proteins, we performed the crosslinking treatment before lysis. Figure 1*B* shows the wild-type Itgα5 clustering along the somite boundary at the experimental stage. Conversely, Itgα5LBD formed no clusters along the somite boundary, while Itgα5AA clustered well along the somite boundary but poorly stabilized on the mesenchymal cell membrane (Fig. 1, *C* and *D*). The activation states and conformations of the two mutant alleles have been confirmed by fluorescence resonance energy transfer-fluorescence lifetime imaging (FRET-FLIM) measurement (30). With these mutant alleles representing inactive and active states of Itgα5, we proceeded to compare the associated protein profiles in each state.

We identified 647 proteins with at least two identified peptides and appearing in at least two biological repeats in one dataset. The full protein and peptide results are listed in supplemental Tables S1 and S2. Of these, 595 were in the Itgα5LBD dataset and 312 in Itgα5AA. We used Intensity-Based Absolute Quantification (iBAQ) for protein quantification. Based on the protein abundance, Itgα5 ranked top and its beta subunits Itgβ1a and Itgβ1b fell not far behind (Fig. 1, *E* and *F*). Itgα5’s primary ECM ligand Fibronectins, Fn1a and Fn1b, ranked in the first half of the Itgα5AA dataset as expected. We also found the ligands in the Itgα5LBD dataset but with protein levels two orders magnitude lower than in the Itgα5AA. This result suggests Itgα5LBD is still in proximity of FN and was thus captured by the crosslinking process. This might be explained by a recent study showing low-affinity integrins bind ligands with faster on- and off-rate compared with the high-affinity conformers (46). We previously reported Cadherin 2 (Cdh2) associates with Itgα5 and represses its activation by stabilizing the inactive conformation (29). Here, we also identify an Itgα5-Cdh2 association in the low end of the distribution for both datasets. We also found Cdh1 on our list, suggesting further possible crosstalk between cell-cell adhesion and cell-ECM interaction. To summarize, our method can pull down Itgα5-associated proteins, including ECM proteins, and those weakly associated with Itgα5 such as Cdh2.

As much of our studies focus on somitogenesis, we also checked the gene annotation at this experimental stage. Full gene expression annotations are listed in supplemental Table S4 and the top 13 terms are plotted in Figure 1*G*. Out of 523 genes (81% of the total) with expression data available, 484 (93%) had gene expression records in structures during the experimental development stages. Of these, besides the half unspecific or whole organism, most genes expressed in the somites (12%), followed by notochord (9%), tail bud (6%), and segmental plate (5%) which includes the presomitic mesoderm. Sub-domains within the nervous system include the immature eye (8%), midbrain (6%), and neural plate (6%). The distributions in the Itgα5LBD and Itgα5AA datasets are similar. Overall, these data suggest Itgα5 and its associated proteins are highly expressed in both tailbud and somites but also in the developing central nervous system.

### Characterizing Activation-State-Dependent Integrin α5 Associated Proteins

To further investigate Itgα5 activation-state-dependent associated proteins, we assigned proteins to the equally shared group Equ, inactive state enriched group LBD, and active state enriched group AA, based on the differential expression analysis. The results are recorded in supplemental Table S3 and plotted in Figure 2*A*. There were 386 proteins in group Equ, 246 in group LBD, and 15 in group AA. We then examined the proteins’ subcellular distribution using GO annotation. The GO annotation and over-representation analysis results are listed in supplemental Tables S4 and S5. As integrins link the ECM and the cytoskeleton, we inspected these related terms in detail (Fig. 2*B*). The AA group presented enrichment in the ECM and collagen-containing ECM. Both the Equ and LBD groups showed marked enrichment in the actin cytoskeleton, actin binding, actin filament binding, and actin filament organization. Overall, these results suggest Itgα5LBD can maintain connections with the actin cytoskeleton which is surprising. Despite the expectation that "inactive" Itgα5LBD would merely reside on the membrane with transient protein contacts with surroundings, it associated with cytoskeletal components comparably to, or even more than, the active Itgα5AA.

We also compared these data with published wild-type zebrafish Itgα5 (Itgα5WT) Co-IP data (30). There were 1012 proteins identified in the WT dataset. In total, 1182 proteins associated with the three Itgα5 alleles: 535 (45%) were unique in Itgα5WT, 98 (8%) in Itgα5LBD, and 27 (2%) in Itgα5AA (supplemental Fig. S3*A*). The unsupervised hierarchical clustering analysis captured a cluster of 824 proteins (70%) with a higher amount in the Itgα5WT compared with the two mutants (supplemental Fig. S3*C*). However, proteins in this cluster were markedly enriched in the nucleolus and RNA processing (supplemental Fig. S3*E*). Clusters with protein levels in Itgα5WT similar to or higher than Itgα5AA recapitulated the ECM-enriched pattern we observed in the grouping analysis (supplemental Fig. S3*D*). Also, clusters with protein levels in Itgα5WT similar to or higher than Itgα5LBD recapitulated the actin cytoskeleton-enriched pattern. Overall, Itgα5AA and Itgα5LBD exhibited representative features of Itgα5 associated proteins. The results captured active Itgα5 engaging in ECM-actin cytoskeleton networks, but surprisingly found the inactive Itgα5LBD also associating with the actin cytoskeleton.

### ECM-Integrin-Actin Network

To examine the networks of proteins along the ECM-integrin-actin axis in more detail, we pulled the proteins through the GO annotations and retrieved the protein-protein interaction (PPI) network (supplemental Table S6 and Fig. 2*C*). We found 37 extracellular proteins, of these, 8 were ECM proteins, including Fibronectin 1a (Fn1a) and collagen type XIV alpha 1 chain (Col14a1a) in the AA group, Fibronectin 1b (Fn1b), Laminins (Lamb1a, Lama1, Lamc1), Cartilage oligomeric matrix protein (Comp), and Fibrillin 2b (Fbn2b) in the Equ, and Apolipoprotein Eb (Apoeb) in the LBD. There were 34 actin cytoskeleton proteins, about two thirds were in group Equ and one third in group LBD. The most abundant protein was Actin, cytoplasmic 1 (Actba/Actb1). In the Equ group, Myosins (Myh9a, Myh10, Myo5ab, Myo6a, Myo5b, Myo1b), and Tropomyosins (Tpm3) were the most prevalent proteins besides Actba. In the LBD group, the most abundant protein was Myosin IC (Myo1ca). We notice that proteins in the LBD group were generally less abundant than in the Equ group on average (Fig. 2*D*). The low pull-down yield may indicate more transient or peripheral associations between Itgα5LBD and these actin proteins.

We next examined cell adhesion and cell junction proteins in the network. These proteins can exhibit crosstalk with integrins and are known to connect to actin (47, 48). We checked whether the pull-down results were biased due to the presence of these proteins. We found in total 26 cell-cell adhesion or junction proteins. Besides Cadherins, there were three Catenins (Ctnnd1, Ctnna1, and Ctnnb1) that can enable cadherin binding activity (49), and three Flotillins (Flot2a, Flot1b, and Flot1a) that are reported to regulate cell-cell adhesion mediated by cadherin (50, 51). Other proteins participating in cell adhesion activities were Protein tyrosine kinase 7a (Ptk7a) and Tight junction proteins (Tjp1b). Although these proteins showed close connections to the actin cytoskeleton, they were in similar or lower abundance than the Cadherins and roughly 2 to 4 magnitude less abundant compared to Itgβ1b. Flotillins were the exception, but they lacked strong connections to other proteins in the network. Thus, the actin cytoskeleton proteins should be mainly linked to Itgα5.

### Ligand-Binding-Deficient Itgα5’s Interactome

As the Itgα5LBD showed surprising engagement with its surroundings, we then took a closer look at its interactome. The results are recorded in supplemental Tables S7, S7.1–S7.3, and plotted in supplemental Fig. S4. Of all well-detected 595 proteins, most localized to the nucleus (37%) and cytoplasm (36%), with secondary localization in the membrane, cytosol, and endoplasmic reticulum, and minor components associated with the actin cytoskeleton (6.3%) and extracellular space (3.6%) (supplemental Fig. S4*B*). Based on protein abundance, we divided the proteins into three classes: high (>75th percentile in intensity rank), medium (25th-75th), and low (<25th). The high class displayed a decreased presence in the cytosol but a significant enrichment in ribonucleoprotein complexes. The low class showed reduced representation in the nucleus and ribonucleoprotein complexes but an enrichment in membrane proteins. The medium class followed the overall distribution patterns but showed reduced representation in membrane proteins. The GO over-representation analysis (supplemental Fig. S4, *C*–*F*) indicated the high and medium classes were significantly enriched in ribosome components, translation machinery, and ribonucleoprotein complex biogenesis, while the medium and low classes were enriched in actin cytoskeletal components.

We then analyzed the PPI network centered on Itgα5β1, focusing on the two clusters: actin cytoskeleton and ribosome (supplemental Fig. S4*G*). Cluster 1 included proteins involved in actin cytoskeleton, actin binding, cell cortex, and anchoring junction, and cluster 2 included ribosome and translation machinery units. There were 51 proteins in cluster 1, comprising 8.6% of the total, and 96 in cluster 2, comprising 16.1%. We stratified the PPI network into four tiers based on the proximity to Itgα5β1. Tier 1 proteins directly interacted with Itgα5β1, and featured medium abundance proteins Ctnnb1, Actn4, Fn1a, Flna, Lamb1a, and Lamc1, and low abundance proteins Cdh2, Iqgap2, Flnb, Cdh1, Lama1, and Itga6a. Among cluster 1, Ctnnb1 was the most abundant in Tier 1, while the highly abundant Actba, Myh9a, and Myh10 were in Tiers 2 and 3. This network's hubs were Cfl1 and Actba with centrality degrees of 0.43 and 0.41, respectively. The connections between Itgα5β1 and the hubs were mediated through Ctnnb1, Actn4, Flna, Flnb, Cdh2, and Cdh1. On the other hand, Itgα5β1 was connected to ribosomal components mainly through Ctnnb1. This cluster started with the Tier 2 proteins Rps6, Rpl19, Ruvbl1, Puf60b, Ruvbl2, and Ybx1. Tier 3 contained both the highest abundant protein Uba52, and the hubs, Rpl7a and Rps16 (centrality degrees = 0.73 and 0.72).

Collectively, these findings suggest Itgα5LBD associated proteins display subcellular heterogeneity by their protein abundance. The links with ribosome clusters might suggest either active Itgα5 synthesis/trafficking or its involvement in ribosome assembly and translational regulation along the Itgα5’s signaling pathway. Although not much about the integrin-ribosome protein interactions has been reported (52), the co-occurrence of integrin abnormalities and enhanced ribosomal protein synthesis, such as increased expression of integrin itself and/or its activator laminins, has been reported in sepsis, tumor progression, and immune responses (53, 54, 55). Itgα5LBD’s interaction with actin cytoskeletal proteins indicates "inactive" integrin might retain the capacity to mediate inside-out signaling.

### Results Validation by PRM

Focusing on the integrin-actin linkers, we next sought to validate the iBAQ results using Parallel Reaction Monitoring (PRM). PRM is a targeted mass spectrometry method with high specificity and efficiency (56, 57). The experimental workflow is illustrated in Figure 3*A*. Briefly, we first built a peptide library from data independent acquisition (DIA) mass spectrometry analysis of total protein extracts from the dissected somites and then selected unique peptides, which serve like molecular fingerprints, for the targets. In the PRM experiment, specific peptide sequences with their mass-to-charge ratio, precursor charge, retention time, etc., were monitored. Peptide and protein quantifications were obtained through subsequent data analysis. Compared with the DDA method used in the discovery phase, which relies on fragmenting high-abundance precursor ions and often overlooks low-abundance proteins, PRM's direct targeting of specific unique peptides improves sensitivity. Also, compared with Western blotting, PRM is antibody-free and enables simultaneous validation of dozens of targets in a single experiment. Therefore, PRM is an efficient and reliable approach to validate our results.

We included three types of proteins. First, Itgα5 and its known interacting proteins, including its beta subunits, Itgβ1a and Itgβ1b, ECM ligands, Fn1a and Fn1b, and the weakly associated protein Cdh2 and putative interacting protein Cdh1. Second, we examined representative proteins in our integrin-actin networks, including Actba, Flna, Actn4, and Cfl1. Third, we examined proteins reported in the integrin-actin networks, such as Talin, Kindlin, etc. Type three proteins were selected mostly based on the adhesome proteins (44) and the consensus list (11), evidence of their expression in somites, and protein detection in the pre-experiment using total protein lysates of the embryos at the 12 to 14 somite stage. We also included the isoforms for some targeted proteins, for instance, Actn1, Actn2b, and Actn3b for Actn4, or Tln1, Tln2a, and Tln2b for Talin. Integrin β5 (Itgβ5) served as a negative control as it was expressed at the 12 to 14 somite stage but was neither detected by iBAQ nor should bind to Itgα5. Details about the targeted protein selections are documented in supplemental Table S12. The quantification results of peptides and proteins are listed in supplemental Tables S10–S12.

First, all of the positive control proteins were detected by PRM (Fig. 3*B*). The presence of Itgβ1a mostly detected in the Itgα5AA dataset displayed the high specificity of PRM as we co-expressed Itgβ1a with Itgα5AA to improve its expression on the cell membrane. One Fibronectin, Fn1b, was also only detected in the Itgα5AA dataset. The remaining targeted peptides of the positive control proteins were found in both datasets. The negative control, Itgβ5, was not detected in either dataset. These data showed good consistency with the iBAQ detection list, suggesting PRM functioned well as a validation tool.

Second, for the integrin-actin linkers, the representative proteins in our networks, Actba, Flna, Actn4, and Cfl1, were all detected by the PRM in both datasets (Fig. 3*C*). In the list of integrin-actin network proteins, we detected Pdlim1 (PDZ and LIM domain 1), Pfn1 (profilin 1), Vaspb (vasodilator stimulated phosphoprotein b), and Pxna (paxillin a). Talins and Kindlins are regarded as the key actin linkers for integrins (58). However, while Talins and Kindlins can be detected in the total protein extracts at the 14 somite stage (supplemental Table S9), we found neither Talins (Tln1, Tln2a, and Tln2b) nor Kindlins (Fermt1 and Fermt2) in the Itgα5 centered networks (Fig. 3*D*). The actin regulator Enah (ENAH actin regulator), adaptors Pdlim 7 (PDZ and LIM domain 7) and Ldb3a (LIM domain binding 3a), and cell adhesion molecule Nexn (nexilin), were also not well detected.

Lastly, we quantified the protein level using the sum of the targeted peptide intensities and made comparisons between the two datasets. We found that Itgα5 was markedly higher in the Itgα5LBD dataset than in the Itgα5AA (*p*-value = 0.003). This observation parallels the reduced membrane stability of the constitutively activated Itgα5AA. However, when normalizing for Itgα5 levels, all the detected proteins were found at markedly higher levels in the Itgα5AA dataset (Fig. 3*E*). This quantification difference between iBAQ and PRM can come from the improved detection specificity and sensitivity of PRM. On the other hand, the Itgα5AA’s cytoplasm retention (Fig. 1*D*) might favor pull down of these actin-linkers under crosslinking treatment. Regardless of this quantification discrepancy, our observation that Itgα5LBD can physically connect to the actin cytoskeleton was confirmed by PRM.

Overall, the PRM methods validated the MS data and suggested a close association between Itgα5 with the actin proteins not only in the active integrin state but also in the inactive state.

### Integrin α5 Associates with α-actinin 4

Given its key position in the Itgα5-actin network, coupled with its bias toward the ligand-binding-deficient allele, Actn4 emerged as a compelling target for further investigation. α-actinin is a family of actin-binding proteins that organize and regulate the actin cytoskeleton (59). α-actinin 4 (Actn4) is the non-muscle-specific member of this family and links integrins and actins through integrin beta subunits, such as Itgβ1, Itgβ2, and Itgβ3 (60, 61, 62, 63). Actn4 competes with other actin linkers, such as Talin, in connecting to integrins during cell adhesion maturation and cell migration (35, 64). With Itgα5, ItgαV, and Itgβ1, Actn4 is one of the integrin adhesome consensus proteins appearing in all seven studies in a meta-analysis (11). However, coupling between Itgα5 and Actn4 has not received much attention. In zebrafish, we detect the association of Actn4 with Itgα5, while other α-actinin isoforms were absent (Fig. 3, *C* and *D*).

The association of Actn4 with Itgα5LBD is unexpected as it is generally thought that only active integrins are linked to the actin cytoskeleton. As a more stringent test of this Actn4-Itgα5LBD association, we performed a PRM experiment on Itgα5LBD pull-down samples without crosslinking. These results are appended to supplemental Tables S10 and S12 and shown in supplemental Fig. S5. In the positive control group of proteins, we found only the Itgβ1a and Itgβ1b. Note the capture of Itgβ1a was likely due to the increased amount of Itgα5 as we increased the starting materials to compensate for the lack of crosslinking. Of all the targeted actin adaptors, Actba, Flna, Actn4, Cfl1, and Pxna were still detected as associated with Itgα5LBD in the absence of crosslinking. Flna and Cfl1 were found to closely resemble the control group, which might be because of their high abundance (supplemental Table S9). Other α-actinin isoforms were absent. These data confirmed the association between Actn4 and Itgα5LBD.

### Integrin α5 and α-actinin 4 Co-Localize at the Zebrafish Somite Boundary

**Fig. 4:**
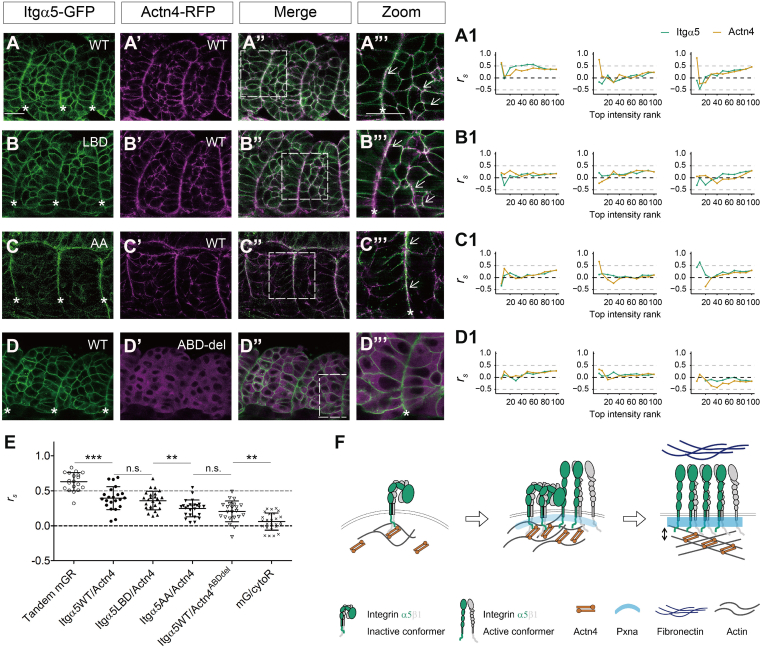
**Integrin α5 and α-actinin 4 co-localize at the zebrafish somite boundary.***A-D*, co-expression of Integrin α5-GFP (*green*) and α-actinin 4 (Actn4-RFP, magenta) in the early zebrafish somites. *A*, Itgα5WT; *B*, Itgα5LBD; *C*, Itgα5AA; *D*, Itgα5WT and actin binding domain deleted Actn4 (Actn4^ABDdel^). Scale bar: 20 μm; size picture: 4X local zoom of the dashed square; *asterisks* indicate somite boundaries; *arrows* indicate co-localization. *A1-D1*, Spearman's Rank Correlation Coefficient (*r*_*s*_) by top intensity rank for somite boundaries in (*A–D*) from *left* to *right*. Note smaller numbers of the top intensity rank denote higher intensities. Dashed lines indicate *r*_*s*_ = −0.5, 0, and 0.5. *E*, summary of *r*_*s*_ along the somite boundaries without intensity rank. Tandem mGR: positive control, tandem membrane-GFP-RFP; mG/cytoR: negative control, co-expression of membrane GFP and cytosol RFP. Data are mean and standard deviation. Dashed lines indicate *r*_*s*_ = 0 and 0.5. Significance level: ∗∗∗ *p*-value <0.0001, ∗∗ *p*-value <0.005, n.s., not significant. Sample size and results are in supplemental Table S14. *F*, illustration of Itgα5 and Actn4 contacts during somite boundary formation.

To follow-up on the proteomics data, we examined the co-localization of Itgα5 and Actn4 in zebrafish somites using confocal microscopy. We co-expressed either Itgα5-GFP, Itgα5LBD-GFP, or Itgα5AA-GFP with Actn4-RFP (Fig. 4). Unlike Itgα5 and Itgα5AA, which showed continuous clustering along the somite boundary, Actn4 showed on-and-off clustering (asterisks in Fig. 4, *A*–*C*). In mesenchymal cells, Actn4 and Itgα5WT/Itgα5LBD also exhibited co-localization at some cell-cell contacts, with marked enrichment at multicellular junctions (arrows in Fig. 4, *A*–*C* Zoom). This is consistent with the idea of Actn4-Itgα5 association before Itgα5 activation. As their clusters co-localized on and off along the somite boundary, we wondered whether the co-localization between the two depends on the level of clustering and then performed a correlation analysis by the intensity ranking. Briefly, for each channel (red and green) along the somite boundary, pixels were selected based on top percentages of fluorescence intensity (5% to 100%), and Spearman's Rank Correlation Coefficient (*r*_*s*_) was calculated between the selected pixels and their matching pixels in the other channel. If their co-localization is cluster-level dependent, we expect a higher *r*_*s*_ at a higher intensity. Expressing the membrane-anchored tandem GFP-RFP (mem-GFP-RFP) as positive control gave the *r*_*s*_ of 0.63 ± 0.13 on average (Fig. 4*E*). This control *r*_*s*_ does not reach the idealized value of 1 because the different maturation times, photobleaching characteristics, and blinking kinetics of the two fluorophores, and possible sample movement during imaging. Co-expressing the mem-GFP and cytosol RFP (cyto-RFP) as negative control gave the *r*_*s*_ of 0.06 ± 0.12 on average. We observed a large variation at top intensities, 5% to 30% (supplemental Fig. S6) which could be due to the increased random error because of the lower number of pixels and thus made the interpretation of the data in this range difficult. From the top 40% intensity onwards, *r*_*s*_ is stable, and we did not see a marked *r*_*s*_ change by the top intensity ranking in either the controls or the experiment data (supplemental Fig. S6, *A*–*E*). Using all the pixels along the boundary, the overall *r*_*s*_ was 0.39 ± 0.16 and 0.36 ± 0.13 for Itgα5WT and Itgα5LBD, significantly higher than 0.25 ± 0.12 for Itgα5AA (*p*-value <0.005, Fig. 4*E*). These results suggest a reduced co-localization between Actn4 and the active conformer Itgα5AA. Although this result was counterintuitive to the first impression from the images, this result is consistent with the MS results in which Actn4's abundance was biased towards Itgα5LBD (Fig. 2*C*). Lastly, we tracked their co-localization over time during somite boundary formation (supplemental Fig. S7). Images were taken every 3 min focusing on the Actn4 clustering along the boundaries. Although at some timepoints, it appears that Actn4 co-clusters with Itgα5, their behavior was generally not highly correlated.

We wondered if it is the actin cytoskeleton rearrangement during the somite boundary formation that brings the Actn4 close to the Itgα5 cytoplasmic domain. Thus, we made an actin binding domain (ABD) truncated Actn4 (Actn4^ABDdel^). This mutant exhibited cytoplasmic localization without enrichment at the cortex (Fig. 4*D*). Correlation analysis between Itgα5 and Actn4^ABDdel^ gave similar results compared to the Itgα5AA and Actn4 (Fig. 4*E*). Note that the magenta channel is a Z projection. The markedly higher *r*_*s*_ in Itgα5/Actn4^ABDdel^ pair than the negative control (*p*-value <0.005, Fig. 4*E*) implies a possible weak association site in Actn4 independent of the ABD domain. Overall, it is likely actin cytoskeleton rearrangement during somite boundary morphogenesis that brings the cross-linker Actn4 in membrane proximity with Itgα5 and not diffusion in the cytosol.

**Fig. 5:**
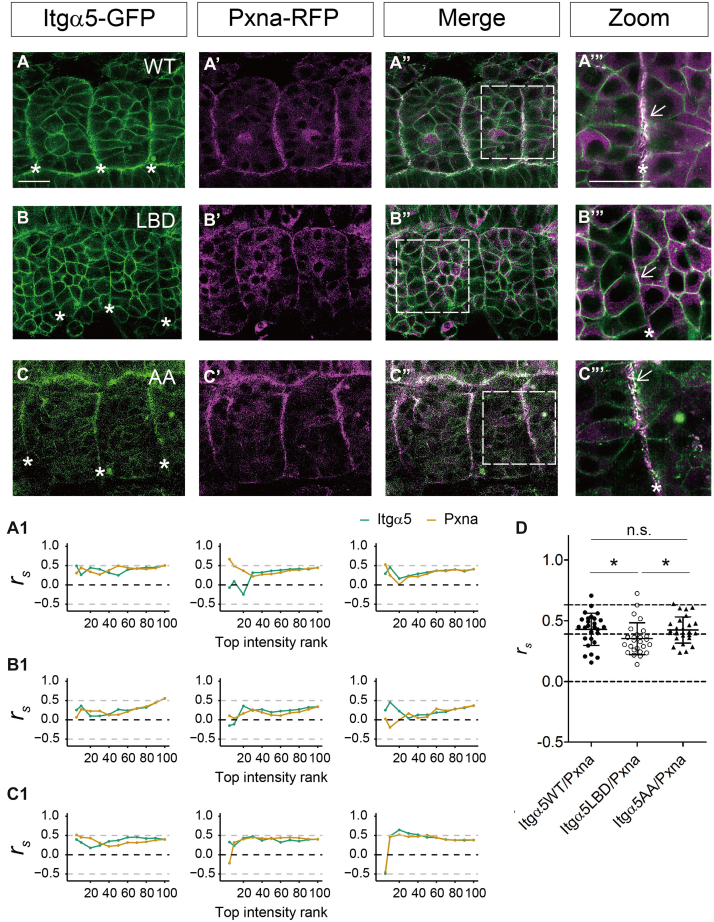
**Integrin α5 and Paxillin a co-localize at the zebrafish somite boundary.***A*–*C*, co-expression of Integrin α5-GFP (*green*) and Paxillin a (Pxna-RFP, magenta) in early zebrafish somites. *A*, Itgα5WT; *B*, Itgα5LBD; *C*, Itgα5AA. Scale bar: 20 μm; size picture: 4X local zoom of the dashed square; asterisks indicate somite boundaries; *arrows* indicate co-localization. *A1-C1*, Spearman's Rank Correlation Coefficient (*r*_*s*_) by top intensity rank for somite boundaries in (*A*–*C*) from *left* to *right*. Note smaller numbers of the top intensity rank denote higher intensities. Dashed lines indicate *r*_*s*_ = 0, 0.39 (Itgα5WT/Actn4), and 0.63 (positive control). *D*, summary of *r*_*s*_ along the somite boundaries without intensity rank. Data are mean and standard deviation. Significance level: ∗ *p*-value <0.05, n.s., not significant. Sample size and results are in supplemental Table S14.

In addition to Actn4, we also found Paxillin a (Pxna) associated with Itgα5LBD using PRM regardless of crosslinking treatment (Fig. 3*C* and supplemental Fig. S5*A*). Pxna is a key adaptor in cell-ECM interaction and functions in focal adhesions and zebrafish somite and myotome development (65, 66, 67). Pxna-RFP exhibited marked accumulation at somite boundaries and a diffuse cytosolic distribution elsewhere (Fig. 5). This is consistent with previous report expressing Pxna-GFP (67). The correlation analysis gave similar *r*_*s*_ for Itgα5/Pxna (0.43 ± 0.13) and Itgα5AA/Pxna (0.42 ± 0.11). Both were also comparable to Itgα5/Actn4 (Fig. 5*D*). In this case, the correlation between Itgα5LBD and Pxna was slightly decreased compared with Itgα5/Pxna and Itgα5AA/Pxna on average (*r*_*s*_ = 0.35 ± 0.13, *p*-value = 0.04 and 0.03, respectively). Time-lapse analysis revealed synchronized clustering of Pxna and Itgα5 during somite boundary formation (supplemental Fig. S8). Accordingly, their correlation over time showed a higher chance of covariance compared with Itgα5/Actn4. The behavior of Pxna is more consistent with our expectation of integrin-actin adaptors in integrin activation. Yet in this case, we also captured Pxna’s covariance and association with Itgα5LBD. Thus, these data provide additional evidence that inactive Itgα5 preserves the capacity to interact with some actin adaptors.

Altogether, our results identify a distinct dynamic association between Itgα5 and Actn4 at the zebrafish somite boundary (Fig. 4*F*). This coupling was characterized by transient contacts rather than stable associations, and Actn4 displayed a preference towards inactive Itgα5 conformers. At the nascent somite boundary, Actn4 might play a role in promoting Itgα5 clustering, as evidenced by the higher *r*_*s*_ remarked for Itgα5WT and Itgα5LBD at the somite boundary, and their colocalization in mesenchymal cells. The similarity between the Itgα5WT and Itgα5LBD suggests a mixture of active and inactive conformers in the somite boundary clusters. The repeated contact and detachment between Itgα5 and Actn4 clusters might help shape boundary cells. As the boundary is established, the contact frequency between Itgα5 and Actn4 declines and Pxna clustering emerges. These data imply a delicate cooperation of Actn4-Itgα5-Pxna in tissue boundary formation.

## Discussion

In this study, we characterized Itgα5 activation-state-dependent associated proteins in zebrafish somites. Our findings revealed the ligand-binding-deficient Itgα5 can connect to the actin cytoskeleton, similar to the constitutively activated allele. Among the integrin-actin linkers, we confirmed the association of the actin cross-linker Actn4 and the actin adaptor Pxna with Itgα5 using PRM. Tracking the Itgα5 and Actn4 clustering along the somite boundary showed their co-localization as an ever-changing feature. Further, experiments suggest that actin-binding is necessary for Actn4 to bind to Itgα5 at the cell membrane. Our study provides a molecular network of Itgα5 in both active and inactive states and offers new insights into integrin activation and integrin-actin interactions *in vivo*.

Integrin-actin adaptors are important in integrin activation and signaling. The focal adhesion network comprises many of these actin adaptors, such as Talin, Kindlin, Vinculin and Paxillin (9, 44). These adaptors typically work in concert, orchestrating integrin activation and signaling in response to the microenvironment. For instance, Talin and Kindlin work together (58). In mechanotransduction, α-actinin competes with Talin to link Integrin β3 with actin to trigger adhesion maturation and transmit force-dependent signaling (35). Here, in the Itgα5-centered network, we found and validated several actin adaptors, including Actba, Flna, Actn4, Cfl1, Pdlim1, Pfn1, Vaspb, and Pxna. Although Talin and Kindlin were detected in the background (supplemental Tables S9), they were not identified as Itgα5 partners in this context (Fig. 3*D*). These results suggest an integrin-specific and/or context-dependent recruitment of the adaptors during integrin activation and signaling.

α-actinins (Actn) are actin cross-linkers, and the non-muscle isoforms (1 and 4) are found in focal contacts and stress fibers (59). α-actinins were found to link integrins to actin more than 30 years ago (60, 61, 68). In integrin-mediated adhesion, Actn1 is the adhesome component whereas Actn4 is the consensus component in a meta-analysis (11, 44). In cell migration, α-actinins enter nascent adhesions periodically and faster than Itgα5, and they are transiently associated in the clusters (69, 70). Similar to this observation, we found that Itgα5 and Actn4 co-localized on and off during the somite boundary morphogenesis. The ligand-binding-deficient Itgα5 can also associate with Actn4, suggesting their association is independent of activation by ligand. In zebrafish, *actn4* is expressed throughout early embryonic development beginning in the oocyte, while the other isoforms’ expression increases after 24 hpf during organogenesis (71). These data suggest a role for Actn4 in integrin function, ECM assembly and tissue morphogenesis during early embryonic development.

The actin cytoskeleton plays a crucial role in tissue morphogenesis, with non-muscle α-actinins regulating cytoskeletal organization. Human ACTN4^K256E^ mutant, associated with a familial form of focal segmental glomerulosclerosis (FSGS), displays enhanced binding affinity to F-actin and causes cytoskeletal “freezing” in podocytes (36, 37). Compared to WT, these mutant proteins are not present in the cell cortex but along stress fibers, and mostly in the Triton-insoluble fraction. We expressed the aligned *D. rerio* Actn4 mutant (Actn4^K245E^) in zebrafish embryos and found similar in-soluble aggregates randomly scattered in cells with ambiguous subcellular localization (supplemental Fig. S9*B*). Phosphomimetic mutations Y4/31E of ACTN4 abolish its actin-binding ability and lead to cytoplasmic retention (38). We also tested this mutant (Actn4^Y4/21E^) but found no change in subcellular localization and a similar correlation with the Itgα5 along the somite boundary comparable to the WT (supplemental Fig. S9, *C* and *D*). Instead, the complete actin binding domain truncated Actn4 (Actn4^ABDdel^) exhibited a diffuse cytoplasmic pattern without cortical actin accumulation and colocalization with Itgα5. Thus, actin-binding by Actn4 recruits Actn4 to Itgα5.

In addition to Actn4, Paxillin (Pxn), a scaffold protein of focal adhesions, was also confirmed to associate with Itgα5LBD even without crosslinking treatment (Fig. 3*C* and supplemental Fig. S5*A*). Pxn mediates the Talin-Kindlin module’s work in focal adhesions, especially in integrin activation and signaling (72, 73). In mechanotransduction, Pxn colocalizes with integrin αVβ3 at high force regions with higher correlation than other adaptors, such as Talin (74). In cell migration, Pxn localizes in cell protrusions earlier than α-actinin and prior to Itgα5 recruitment (69). In zebrafish, Pxn is not only localized strongly along the somite boundary but also plays a key role in somite and myotome development (66, 67). Here, we observe the association between Paxillin a (Pxna) and Itgα5 along the somite boundary (Fig. 5). Our results suggest an Actn4-Itgα5-Pxna cooperation in Itgα5 activation and tissue boundary formation.

The absence of well-characterized integrin–actin adaptor modules, such as talins and kindlins, in this Itgα5-centered network was unexpected. In total protein extracts from 14 somite stage embryos, we detected 53% of the adhesome and 78% of the consensus (supplemental Table S15 and supplemental Fig. S10). These included modules like Talin-Kindlin-Vinculin (Tln1/Tln2a/Tln2b-Fermt1/Fermt2-Vcla/Vclb) and ILK-PINCH-Parvin (Ilk-Lims1-Parvaa), and key components like Tensin (Tns1b/Tns2b), Focal Adhesion Kinase (Ptk2ab/Ptk2bb/Ptk7a), and Ponsin (Sorbs3) (11, 14). However, only 10% and 22% of these proteins were captured in the Itgα5 network under the crosslinking treatment, and more than half were highly abundant proteins in the total protein extracts. Thus, one reason for their absence is their low expression levels, for instance, in the case of the Ilk-Lims1-Parvaa complex. On the other hand, since the crosslinking treatment can capture transient and weak associations, highly and moderately expressed candidates should not be easily missed. In the PRM experiment with improved sensitivity, we found 4 out of 13 candidates missed in the discovery dataset (Fig. 3, *C* and *D*). Talins and kindlins were at medium expression levels similar to those of background Itgα5 or Pxna. However, they were missing not only in the Itgα5WT and Itgα5LBD datasets but also in the constitutively activated Itgα5AA dataset. The discrepancies in the candidate recruitment between the adhesome and our Itgα5 Co-IPs may arise from the spatial and temporal scale differences between focal adhesions and ECM assembly. Overall, this study reveals an integrin and context dependent network preference. Our data are valuable resources for employing zebrafish somites as an *in vivo* platform to study integrin activation.

Integrin trafficking is an important process that regulates the spatiotemporal distribution and activity of integrins on the cell surface (75, 76). We found three Flotillins (Flot2a, Flot1b, Flot1a) with high abundance in the mass spectrometry data (Fig. 2*C*). Flotillins (Flots) anchor to the cytoplasmic leaflet of membranes and are considered lipid raft components. They participate in clathrin-independent endocytosis, and function in membrane receptor clustering (77, 78). Flots stabilize cadherins (both N- and E-cadherins) at cell-cell contacts (50). Our results showed both Cadherins associated with Itgα5 under crosslinking treatment but at a much lower level compared with Flots. Thus, the co-immunoprecipitated Flots are likely Itgα5 dependent. Indeed, Flots recycle Integrin α5β1 to focal adhesions (79), and knockdown of Flots affects focal adhesion formation and integrin signaling (80). Interestingly, Flots and α-actinins colocalize and display FRET at the cell periphery in cell migration (80). In zebrafish embryos, *flot2a* and *flot1b* localize along the somite boundary (81). Morpholino knock-down of Flots causes an E-cadherin accumulation defect at cell-cell contacts of deep cells and thus leads to delayed epiboly development in zebrafish (51). *flot2* morphants also display aberrant somite development. Altogether, these data suggest, as a candidate of integrin partners, Flots might engage in integrin-cadherin crosstalk and integrin-actinin association. Flots’ roles in Itgα5 activation, endocytosis, and ECM assembly merit further investigation.

There are several caveats to consider in this study. First, although from the ligand-binding-deficient properties and its closed conformation shown by FRET-FLIM (30), Itgα5LBD is inactive by default in terms of outside-in signaling, it is not necessarily inactive in mediating the inside-out signaling. As shown by our results, Itgα5LBD preserves the connections to the actin cytoskeleton with an ability similar to that of the constitutively activated allele. Second, the influence of endogenous proteins needs to be considered. While our initial experiments were conducted in MZItgα5^−/−^ embryos to exclude endogenous Itgα5 effects, subsequent validation experiments were performed in wild-type embryos. The presence of endogenous Itgα5 can allow proteins that interact with wild-type proteins to be captured by Itgα5LBD. On the other hand, given the dimeric nature of Actn4, our observations from expressing RFP tagged Actn4 or its mutant may represent a mixture of hybrids with the wild-type protein and the pure homodimers, thus compromising the conclusions. It would be better to test these associations in the absence of background Itgα5 and Actn4. Nevertheless, our discovery dataset and the intensity-based covariance analysis suggests a preferential association between Itgα5LBD and Actn4 compared with Itgα5AA. This bias may result from the activated Itgα5AA recruiting more downstream proteins, such as Pxna, which could spatially compete with or prevent Actn4 from approaching. Further investigation with higher spatiotemporal resolution techniques is needed to elucidate the underlying molecular mechanisms.

Many studies on integrin activation are performed using cell culture and focus on cell migration and focal adhesions. Here, we use the zebrafish somite as the context to explore Itgα5 associated proteins, and thus provide more details of integrin networks under physiological conditions. We used PRM to validate the coarse MS results (57, 82). Given early zebrafish embryos' limited material, PRM offers an antibody-free and high-efficiency method that enables simultaneous validation of multiple proteins in a single experiment. This is especially useful to distinguish family isoforms which are common in zebrafish. In summary, we found that Itgα5 in the ligand-binding-deficient state enriched actin adaptors *in vivo*. These results provide evidence of integrin inside-out signaling under physiological conditions and suggest the role of integrin-actin adaptors interaction in morphogenesis.

## Data Availability

For label-free mass spectrometry, the mass spectrometry proteomics data have been deposited to the ProteomeXchange Consortium via the PRIDE (83) partner repository with the dataset identifier PXD024942 and PXD065495.

For DIA experiment, the mass spectrometry proteomics data have been deposited to the ProteomeXchange Consortium via the PRIDE partner repository with the dataset identifier PXD058516.

For PRM experiments, the mass spectrometry proteomics data have been deposited to the ProteomeXchange Consortium via the PRIDE partner repository with the dataset identifier PXD058550. Skyline files have been deposited to PanoramaWeb https://panoramaweb.org/UUff2q.url and associated with the ProteomeXchange identifier PXD058747.

Public access will become available once the article is accepted.

## Supplemental Data

This article contains supplemental data.

## Conflict of Interest

The authors declare that there are no conflicts of interest with the contents of this article.

## Supplementary data

Video_S2

Supplemental Data Resubmit 2
