# Supplementary material for: Actn4 Links Inactive Integrin α5 With Actin in Zebrafish Somites
Source: Mol Cell Proteomics. 2025 Oct 8;25(2):101087. doi: 10.1016/j.mcpro.2025.101087 (PMC12860946; doi:10.1016/j.mcpro.2025.101087)
Supplement: Supplemental Table S15.1 [file mmc19.docx]

Supporting information text

Supplemental Video S1. Time-lapse of co-expression of Itga5-GFP (green) and Actn4-RFP (magenta). Time-lapses were taken at 3 minute intervals.

Supplemental Video S2. Time-lapse of co-expression of Itga5-GFP (green) and Pxna-RFP (magenta). Time-lapses were taken at 3 minute intervals.
