# Supplementary material for: Actn4 Links Inactive Integrin α5 With Actin in Zebrafish Somites
Source: Mol Cell Proteomics. 2025 Oct 8;25(2):101087. doi: 10.1016/j.mcpro.2025.101087 (PMC12860946; doi:10.1016/j.mcpro.2025.101087)
Supplement: Supporting Information Text [file mmc20.pdf]

Actn4 links inactive Integrin α5 with actin in zebrafish somites

Guangyu Sun[1,2]*, Scott A. Holley[2]*

1. Xiamen Cardiovascular Hospital of Xiamen University, School of Medicine, Fujian Branch of National Clinical Research Center for Cardiovascular Diseases, Xiamen, Fujian 361000, China.

2. Department of Molecular, Cellular and Developmental Biology. Yale University, 260 Whitney Ave, New Haven, CT 06520, USA.

*Corresponding Author: sunguangyu@xmu.edu.cn; scott.holley@yale.edu

Supplemental FIG. 1. **Data reproducibility**.

Supplemental FIG. 2. **Representative samples of Coomassie staining**.

Supplemental FIG. 3. **Comparison with wild-type Itgα5.**

Supplemental FIG. 4. **Itgα5LBD interactome.**

Supplemental FIG. 5. **Parallel Reaction Monitoring (PRM) validates Itgα5LBD associated proteins without crosslinking treatment.**

Supplemental FIG. 6. **Protein localization correlation analysis by top fluorescence intensity rank.**

Supplemental FIG. 7. **Contact dynamics between Itgα5 and Actn4 during Actn4 clustering build-up at the zebrafish somite boundary.**

Supplemental FIG. 8. **Contact dynamics between Itgα5 and Pxna during clustering build-up at the zebrafish somite boundary.**

Supplemental FIG. 9. **Actn4 alleles.**

Supplemental FIG. 10. **PPI network of focal adhesion components in early zebrafish somites.**

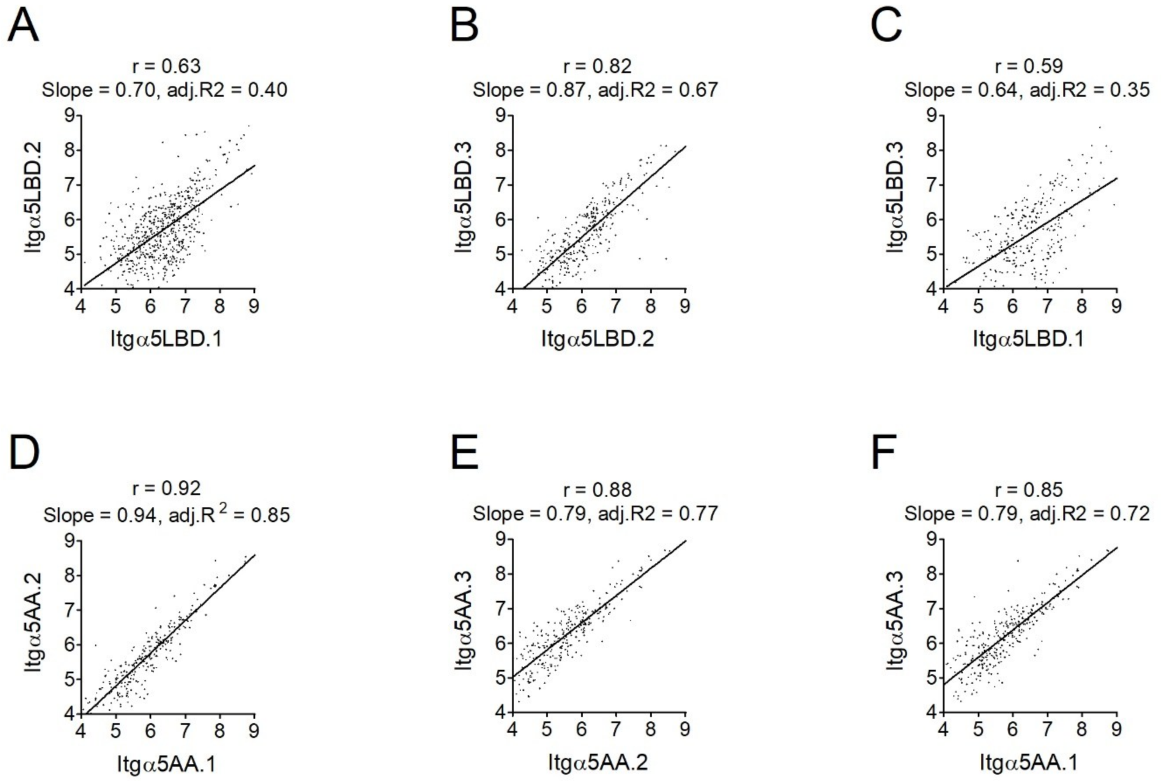

Supplemental FIG. 1. **Data reproducibility**. *r*, Pearson's correlation coefficient; slope, linear regression; adj.R2, adjusted R square. Only proteins present in both replicates were used for analysis.

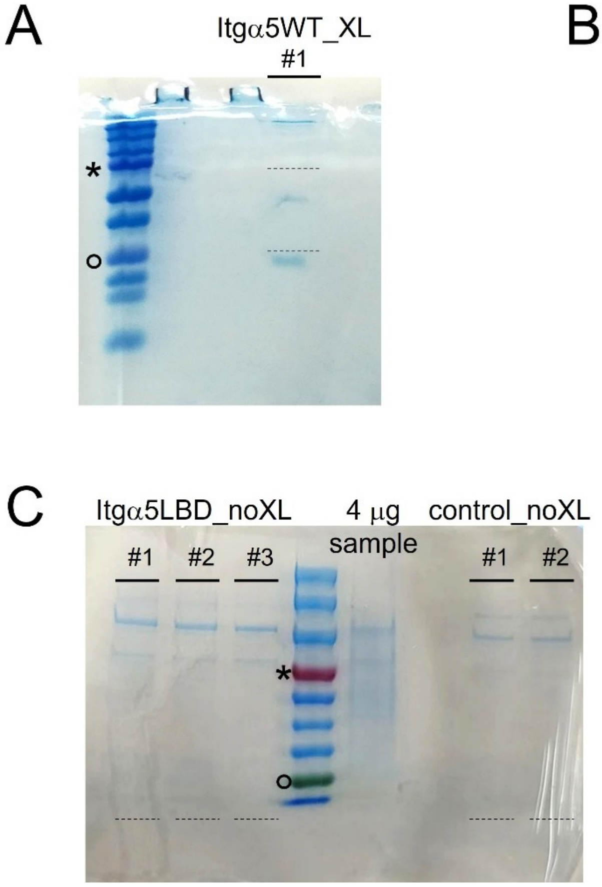

Supplemental FIG. 2. **Representative samples of Coomassie staining**. A and B, Itgα5WT with crosslinking (XL) treatment using a homemade Coomassie staining/destaining buffer. C, Itgα5LBD and control without crosslinking (noXL) treatment using a commercial Coomassie staining buffer. Dashed lines indicate the gel cutting positions. Asterisks represent 75 kDa molecular weight markers and circles represent 25 kDa.

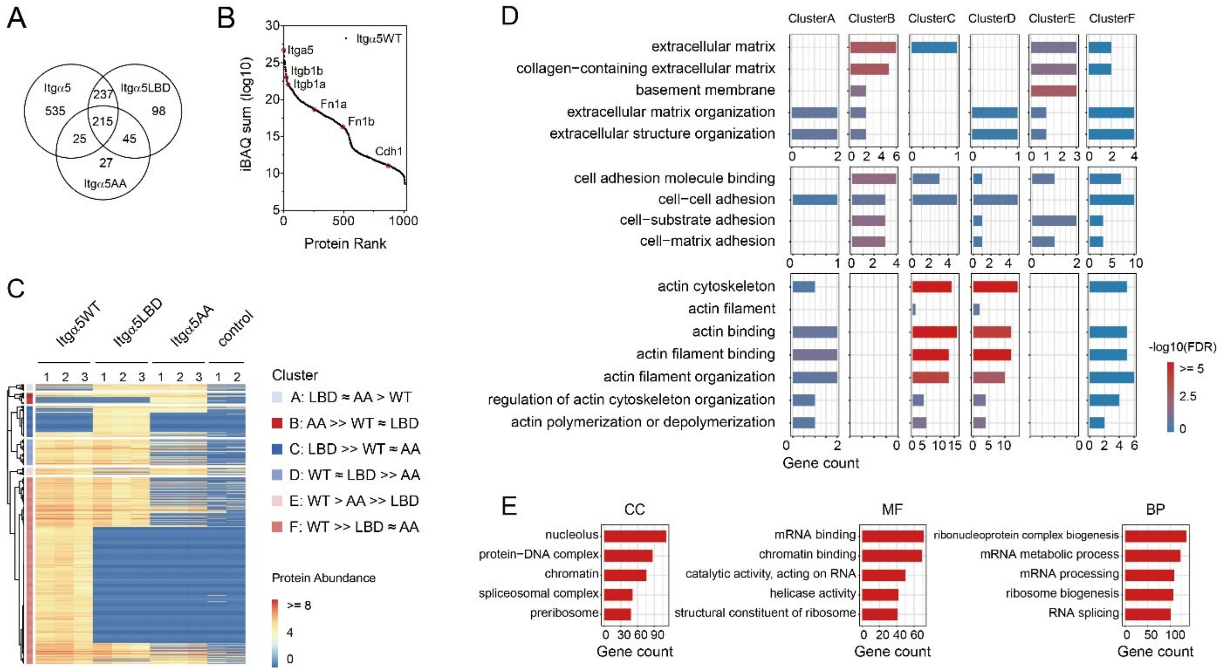

Supplemental FIG. 3. **Comparison with wild-type Itgα5.** A, Venn diagram. B, protein abundance rank in Itgα5WT. Red circle: representative proteins with protein symbols shown. C, unsupervised hierarchy clustering analysis. Color code: protein abundance in log10 transform. D, over-representation analysis of the representative ECM, cell adhesion, and actin cytoskeleton GO terms by clusters. Color code indicates FDR. E, representative GO terms of cluster F. Same color code with figure D. Results are in supplemental Tables S1 and S5.

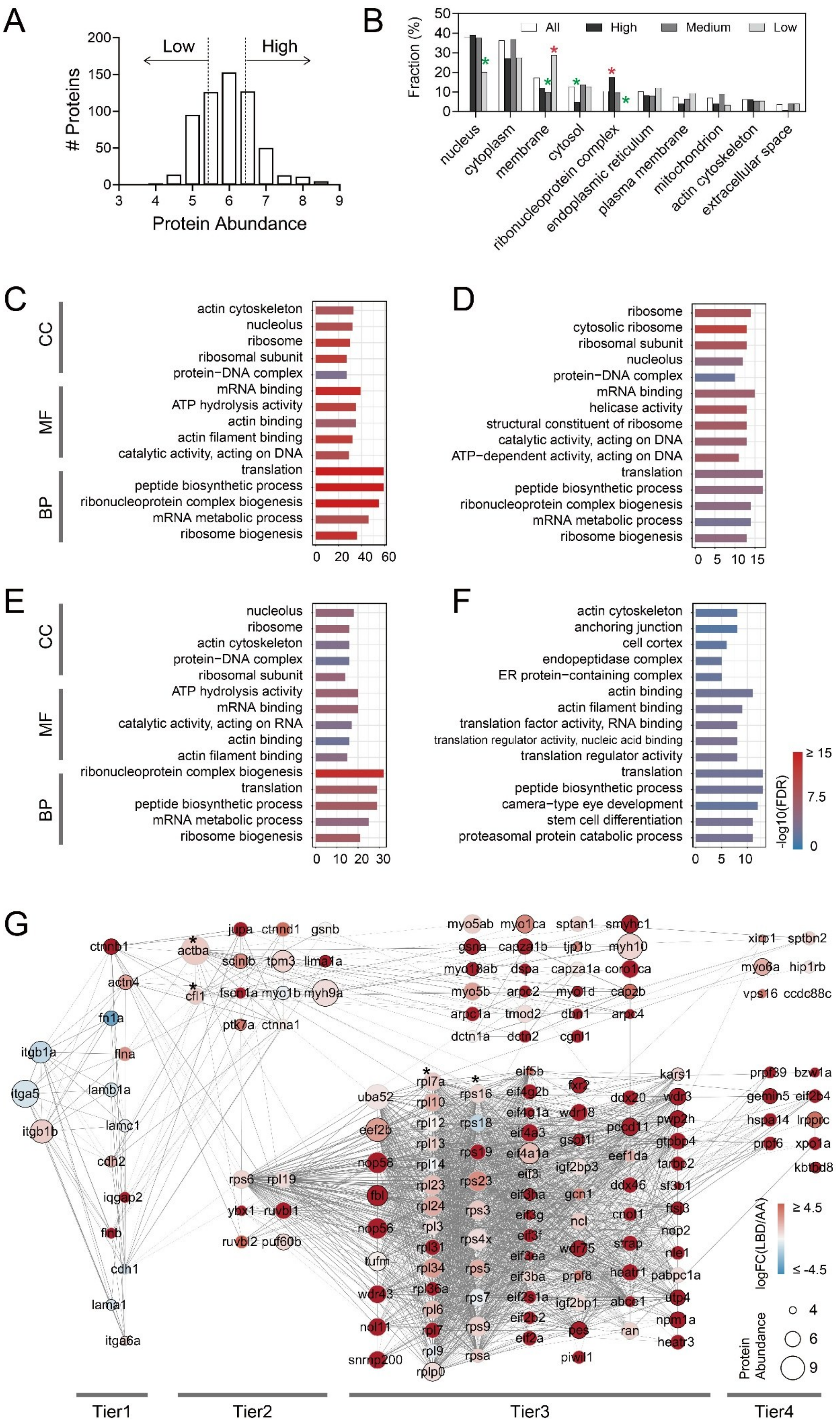

A
Proteins
Low
High
Protein Abundance
B
Fraction (%)
All
High
Medium
Low
nucleus
cytoplasm
membrane
cytosol
ribonucleoprotein complex
endoplasmic reticulum
plasma membrane
mitochondrion
actin cytoskeleton
extracellular space
C
CC
MF
BP
actin cytoskeleton
nucleolus
ribosome
ribosomal subunit
protein−DNA complex
mRNA binding
ATP hydrolysis activity
actin binding
actin filament binding
catalytic activity, acting on DNA
translation
peptide biosynthetic process
ribonucleoprotein complex biogenesis
mRNA metabolic process
ribosome biogenesis
D
ribosome
cytosolic ribosome
ribosomal subunit
nucleolus
protein−DNA complex
mRNA binding
helicase activity
structural constituent of ribosome
catalytic activity, acting on DNA
ATP−dependent activity, acting on DNA
translation
peptide biosynthetic process
ribonucleoprotein complex biogenesis
mRNA metabolic process
ribosome biogenesis
E
CC
MF
BP
nucleolus
ribosome
actin cytoskeleton
protein−DNA complex
ribosomal subunit
ATP hydrolysis activity
mRNA binding
catalytic activity, acting on RNA
actin binding
actin filament binding
ribonucleoprotein complex biogenesis
translation
peptide biosynthetic process
mRNA metabolic process
ribosome biogenesis
F
actin cytoskeleton
anchoring junction
cell cortex
endopeptidase complex
ER protein−containing complex
actin binding
actin filament binding
translation factor activity, RNA binding
translation regulator activity, nucleic acid binding
translation regulator activity
translation
peptide biosynthetic process
camera−type eye development
stem cell differentiation
proteasomal protein catabolic process
-log10(FDR)
≥ 15
7.5
0
G
logFC(LBD/AA)
≥ 4.5
≤ -4.5
Protein Abundance
Tier1
Tier2
Tier3
Tier4

Supplemental FIG. 4. **Itgα5LBD interactome.** A, protein abundance distributions of the Itgα5LBD associated proteins. Protein abundance classes are categorized based on quantitative ranking: “High” is above the 75th percentile, “Low” is below the 25th percentile, and “Medium” is between the 25th and 75th percentiles. B, subcellular distributions of Itgα5LBD associated proteins. Red asterisks represent a significantly increased ratio compared to the whole dataset (All) and green ones represent a significantly decreased ratio. C-F, top 5 enriched GO terms in CC, MF, and BP for the whole dataset (C), High (D), Medium (E), and Low (F) abundance proteins. Color code indicates FDR. ER: endoplasmic reticulum. G, PPI network of the Itgα5LBD associated proteins in actin cytoskeleton and ribosome related GO terms. Gene names are used for illustration. Node size: protein abundance using the median value of the three replicates in log10 transform; node color: fold change (FC); edge thickness: interaction score; node with a black circle: gene expression in the somite area**.** Tier 1 represents direct interactions with Itgα5β1, Tier 2 represents secondary interactions requiring one intermediate protein to connect with Itgα5β1, and so on. Tier 4 also covers proteins without any contact with Itgα5β1. Asterisks indicate hub proteins. Results are in supplemental Tables S7 and S7.1-7.3.

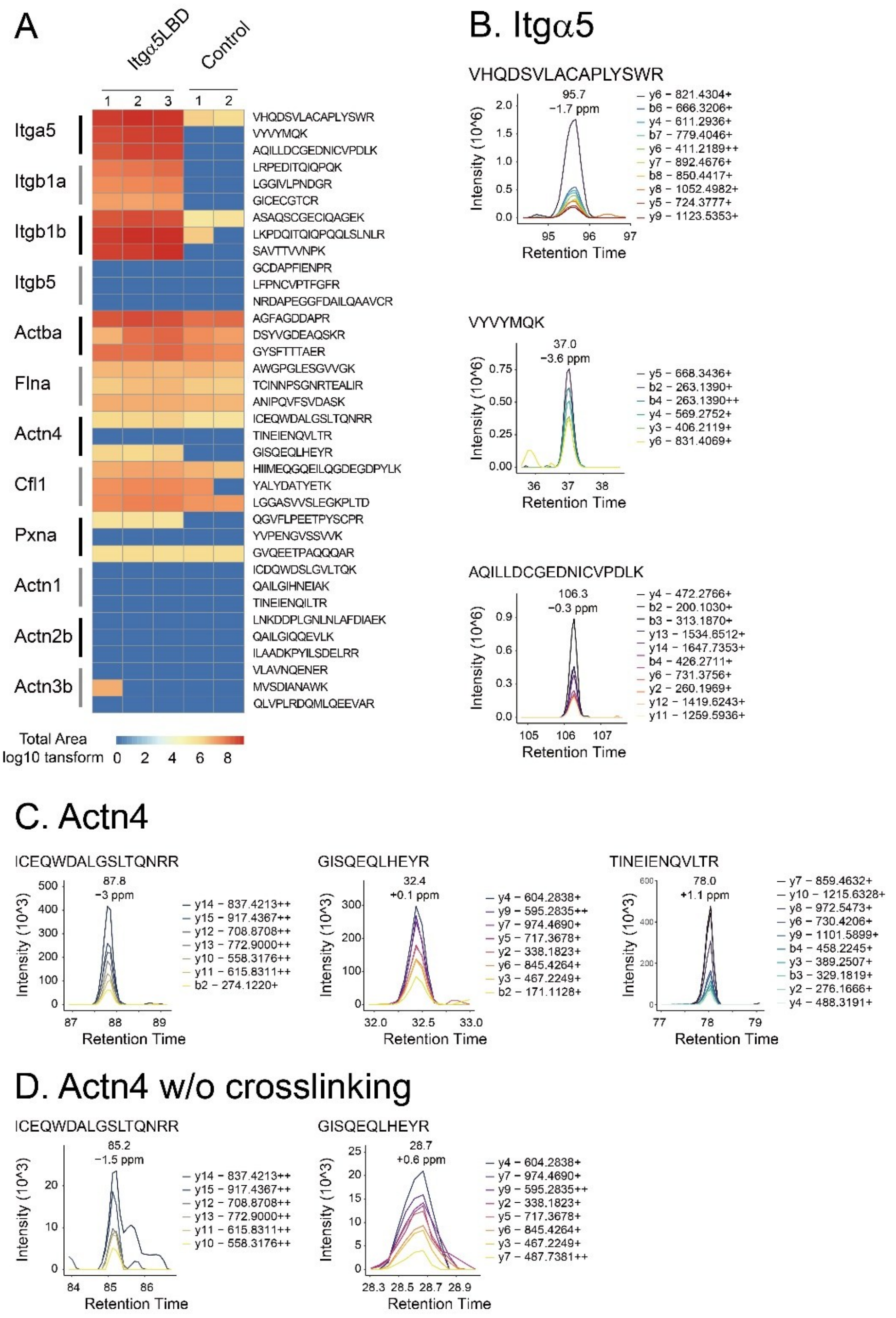

Supplemental FIG. 5. **Parallel Reaction Monitoring (PRM) validates Itgα5LBD associated proteins without crosslinking treatment.** A, selected PRM results. Results are in supplemental Table S11. B-D, precursor profiles of Itgα5 (B) and Actn4 (C and D).

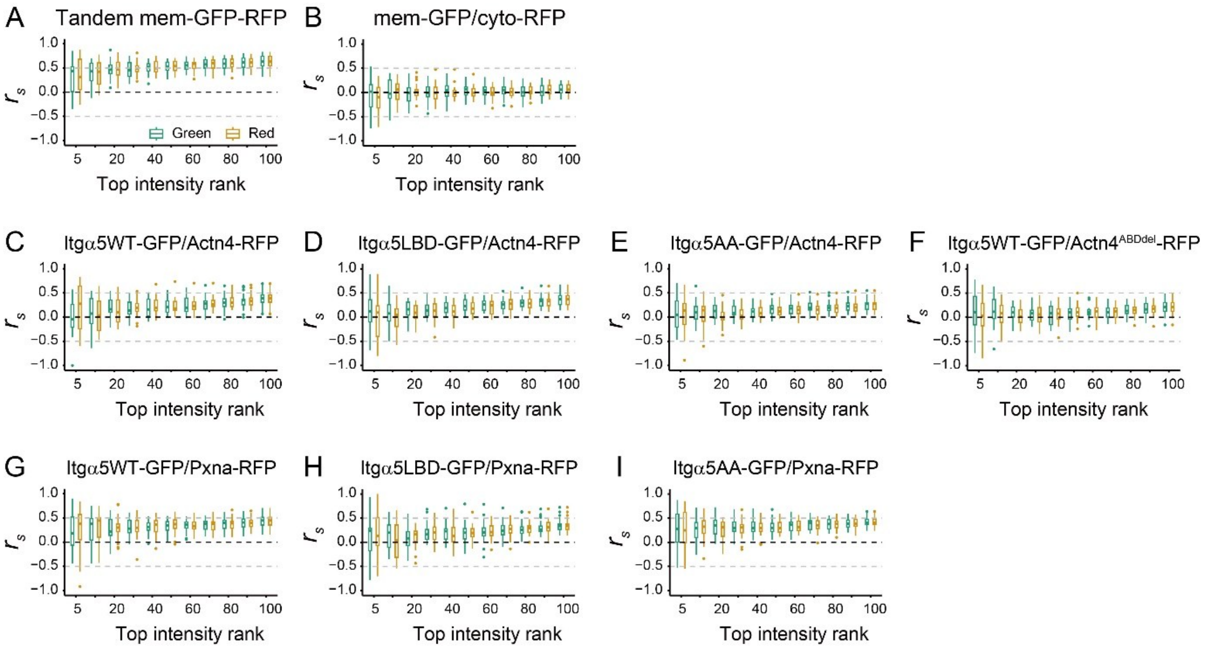

Supplemental FIG. 6. **Protein localization correlation analysis by top fluorescence intensity rank.** Spearman's Rank Correlation Coefficient (r_s) statistics by top intensity rank of positive control tandem mem-GFP-RFP (A), negative control mem-GFP/cyto-RFP (B), Itgα5WT/Actn4 (C), Itgα5LBD/Actn4 (D), Itgα5AA/Actn4 (E), Itgα5WT/Actn4^{ABDdel} (F), Itgα5WT/Pxna (G), Itgα5LBD/ Pxna (H), Itgα5AA/ Pxna (I). At least 3 embryos were used for each dataset. Data are medians and interquartile ranges. Dashed lines indicate r_s = -0.5, 0, and 0.5. Note smaller numbers of the top intensity rank denote higher intensities.

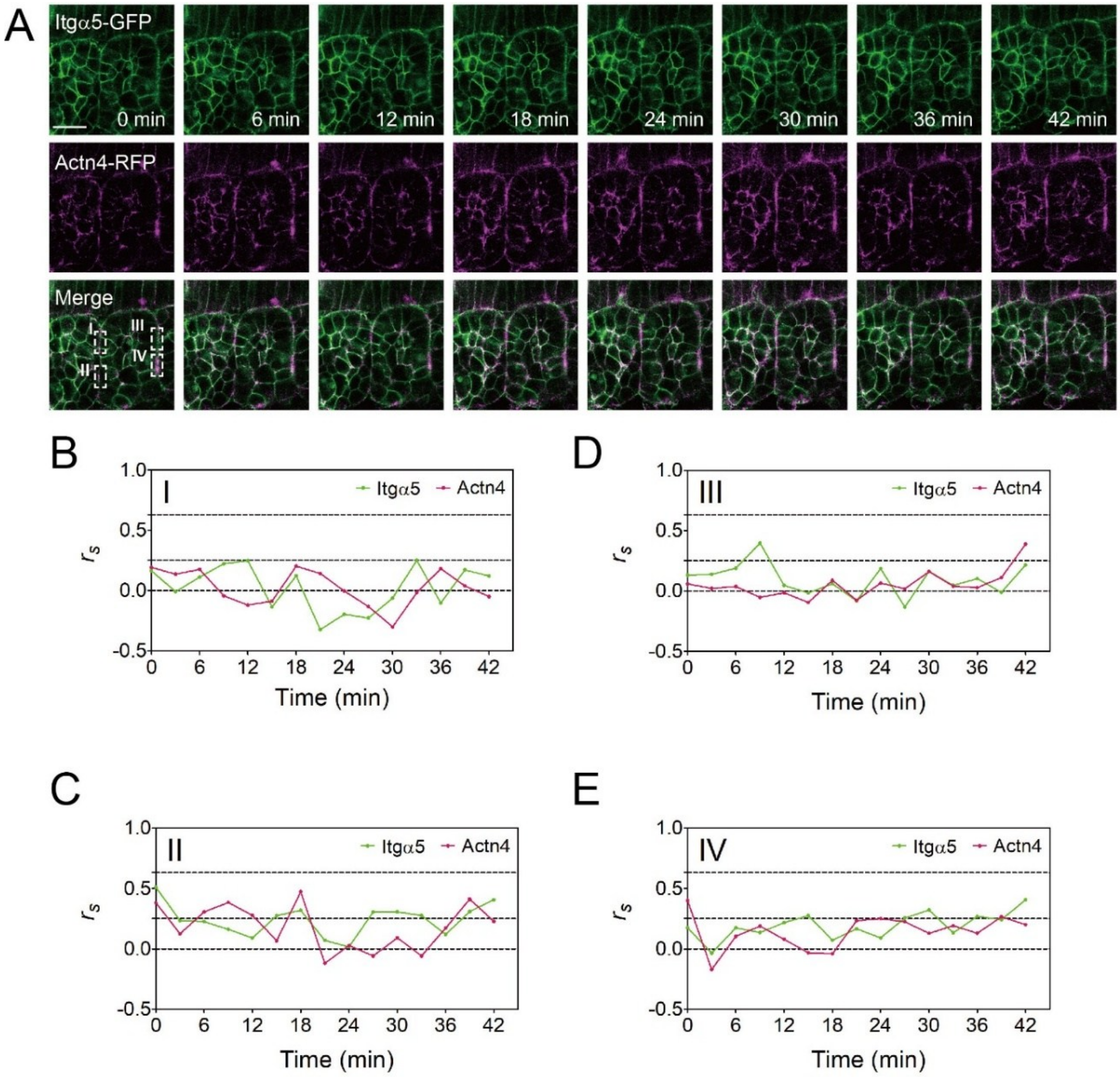

Supplemental FIG. 7. **Contact dynamics between Itgα5 and Actn4 during Actn4 clustering build-up at the zebrafish somite boundary.** A, time-lapse of co-expression of Itgα5-GFP (green) and Actn4-RFP (magenta). Scale bar: 20 μm. For simplicity, the image series shows 6 minute intervals, and the full time-lapse is supplemental video 1. B-E, correlation analysis of ROIs, dashed square I-IV in A, using Itgα5 (green) or Actn4 (magenta) as reference. Dashed lines indicate r_s = 0, 0.25 (Itgα5AA/Actn4), and 0.63 (positive control).

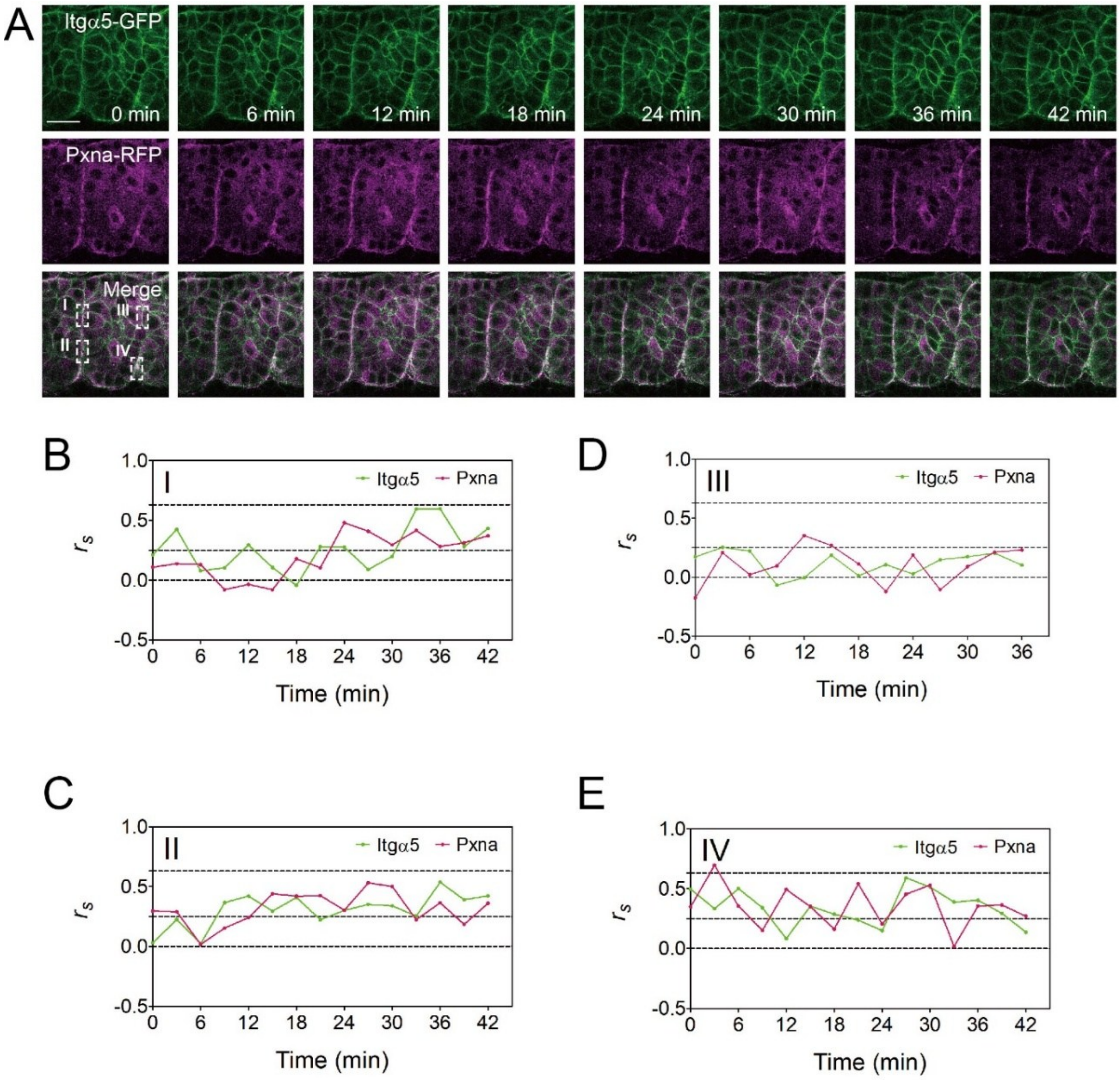

Supplemental FIG. 8. **Contact dynamics between Itgα5 and Pxna during clustering build-up at the zebrafish somite boundary.** A, time-lapse of co-expression of Itgα5-GFP (green) and Pxna-RFP (magenta). Scale bar: 20 μm. For simplicity, the image series shows 6 minute intervals, and the full time-lapse is supplemental video 2. B-E, correlation analysis of ROIs, dashed square I-IV in A, using Itgα5 (green) or Pxna (magenta) as reference. Dashed lines indicate r_s = 0, 0.25 (Itgα5AA/Actn4), and 0.63 (positive control).

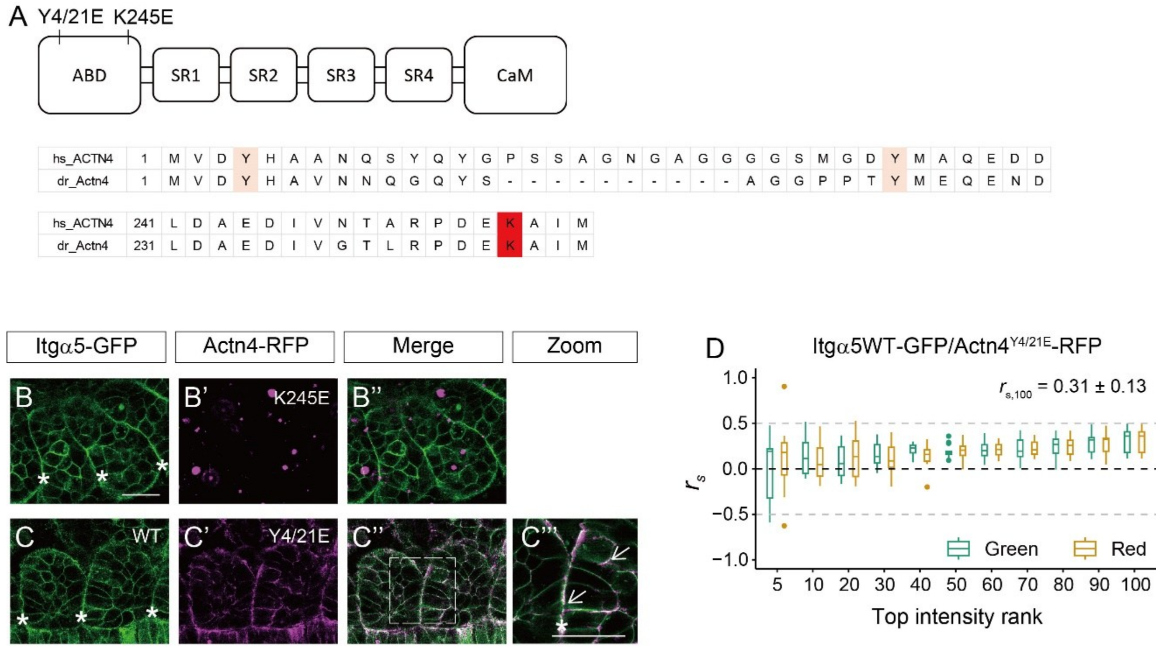

Supplemental FIG. 9. **Actn4 alleles.** A, point mutations (shaded) are indicated in an alignment of Danio rerio (dr) Actn4 with homo sapiens (hs) orthologous ACTN4. B-C, co-expression of Itgα5-GFP and Actn^{K245E}-RFP (B) or Actn4$^{Y4/21E}$-RFP (C). Scale bar: 20 μm; size picture: 4X local zoom; asterisks indicate somite boundary; arrows indicate Itgα5 and Actn4 co-localization. D, Spearman's Rank Correlation Coefficient (r_s) statistics by top intensity rank of Itgα5WT/ Actn4$^{Y4/21E}$. Three embryos were analyzed. Data are medians and interquartile ranges. Dashed lines indicate r_s = -0.5, 0, and 0.5.

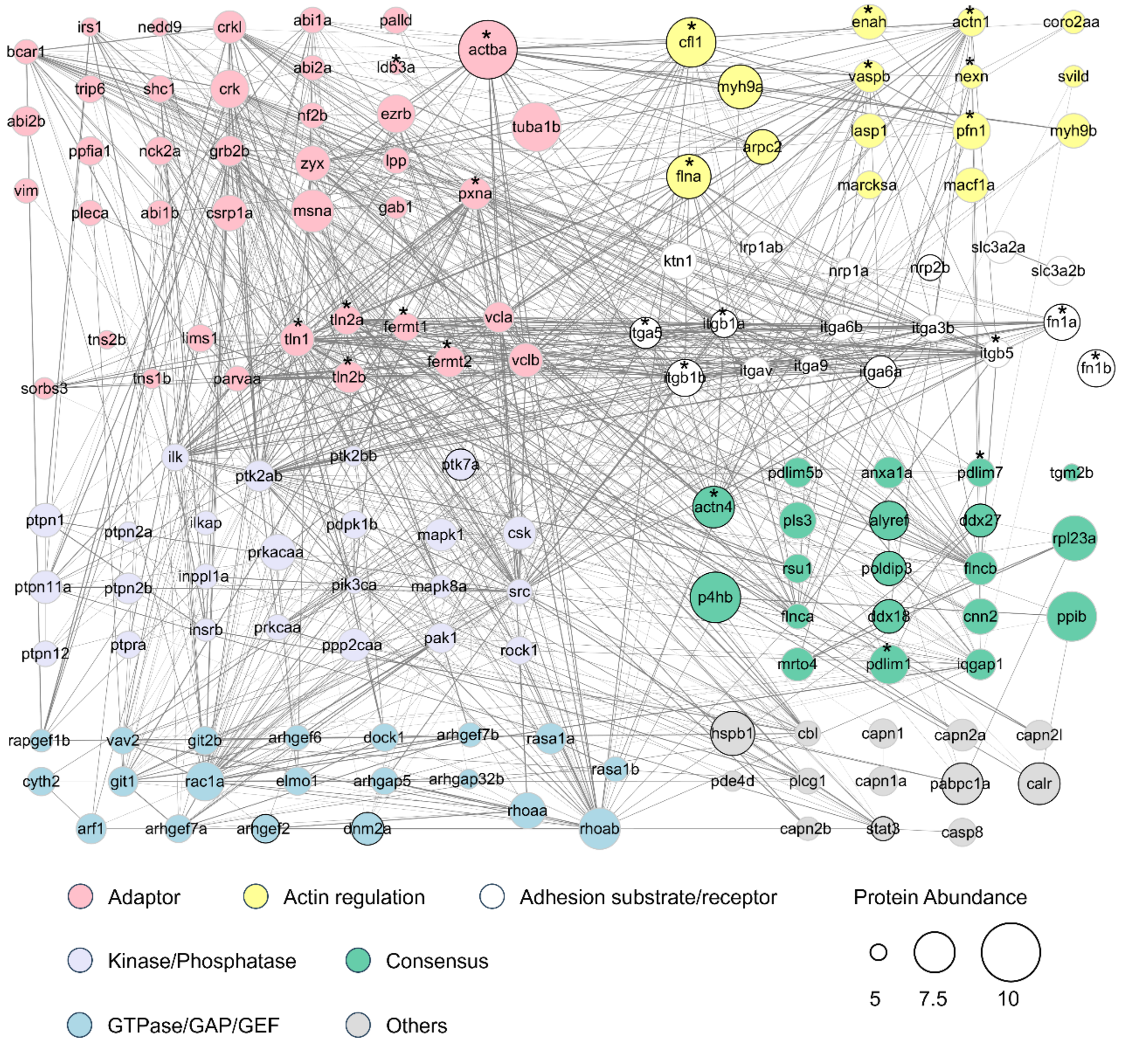

Supplemental FIG. 10. **PPI network of focal adhesion components in early zebrafish somites.** Color code: functional categories from the adhesome (https://adhesome.org/components.html). For simplicity, Kinase/Phosphatase includes tyrosine kinase, serine/threonine kinase, serine/threonine phosphatase, PtdIns phosphatase, PtdIns kinase, and tyrosine phosphatase; Others includes chaperone, protease, E3-ligase, RNA or DNA regulation, cAMP phosphodiesterase, phospholipase, channel, and serine palmitoyltransferase. The Consensus category are the consensus proteins that are not listed in the adhesome. Node size: protein

abundance using the median value of the three replicates in log10 transform. Asterisks: protein candidates in PRM experiments. Node with a black circle: protein detected in Itgα5 Co-IPs. Data are DIA results at the 14 somite stage. Note that Tln2a, Tln2b, Tns2b, and Ptk7a are not in any adhesome or consensus list but are captured by the protein name. Results are in supplemental Tables S9, S15, and S15.1.
